# Identification of 2-Aminoacyl-1,3,4-thiadiazoles as Prostaglandin E_2_ and Leukotriene Biosynthesis Inhibitors

**DOI:** 10.1021/acsmedchemlett.2c00343

**Published:** 2022-12-09

**Authors:** Marianna Potenza, Assunta Giordano, Maria G. Chini, Anella Saviano, Christian Kretzer, Federica Raucci, Marina Russo, Gianluigi Lauro, Stefania Terracciano, Ines Bruno, Maria Iorizzi, Robert K. Hofstetter, Simona Pace, Francesco Maione, Oliver Werz, Giuseppe Bifulco

**Affiliations:** † Department of Pharmacy, University of Salerno, via Giovanni Paolo II, 132, 84084, Fisciano, Italy; ¶ The FIRC Institute of Molecular Oncology, Via Adamello 16, 20139 Milan, Italy; § Institute of Biomolecular Chemistry (ICB), Consiglio Nazionale delle Ricerche (CNR), Via Campi Flegrei 34, Pozzuoli, 80078 Napoli, Italy; ∥ Department of Biosciences and Territory, University of Molise, Contrada Fonte Lappone, Pesche, 86090 Isernia, Italy; ⊥ ImmunoPharmaLab, Department of Pharmacy, School of Medicine and Surgery, University of Naples Federico II, Via Domenico Montesano 49, 80131 Naples, Italy; # Department of Pharmaceutical/Medicinal Chemistry, Institute of Pharmacy, Friedrich Schiller University Jena, Philosophenweg 14, 07743 Jena, Germany

**Keywords:** Combinatorial virtual screening, 2-Aminoacyl-1,3,4-thiadiazole, mPGES-1, Leukotriene biosynthesis pathway, Anti-inflammatory activity

## Abstract

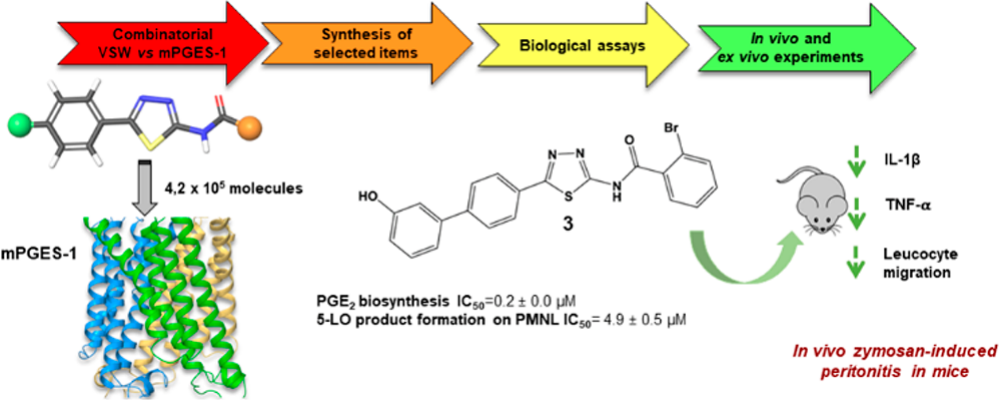

The application of a multi-step scientific workflow revealed an unprecedented class of PGE_2_/leukotriene biosynthesis inhibitors with *in vivo* activity. Specifically, starting from a combinatorial virtual library of ∼4.2 × 10^5^ molecules, a small set of compounds was identified for the synthesis. Among these, four novel 2-aminoacyl-1,3,4-thiadiazole derivatives (**3**, **6**, **7**, and **9**) displayed marked anti-inflammatory properties *in vitro* by strongly inhibiting PGE_2_ biosynthesis, with IC_50_ values in the nanomolar range. The hit compounds also efficiently interfered with leukotriene biosynthesis in cell-based systems and modulated IL-6 and PGE_2_ biosynthesis in a lipopolysaccharide-stimulated J774A.1 macrophage cell line. The most promising compound **3** showed prominent *in vivo* anti-inflammatory activity in a mouse model, with efficacy comparable to that of dexamethasone, attenuating zymosan-induced leukocyte migration in mouse peritoneum with considerable modulation of the levels of typical pro-/anti-inflammatory cytokines.

Microsomal prostaglandin E_2_ synthase-1 (mPGES-1),^1^ a downstream PG synthase, is a membrane-integrated protein able to convert the cyclooxygenase (COX)-derived unstable prostaglandin H_2_ (PGH_2_) to the bioactive prostaglandin E_2_ (PGE_2_). This enzyme is one of the membrane-associated proteins involved in the metabolism of glutathione and prostanoids (MAPEG), a family of proteins including several key targets, such as the 5-lipoxygenase-activating protein (FLAP), leukotriene C_4_ synthase (LTC_4_S), and microsomal glutathione S-transferases, useful for the development of anti-inflammatory and anticancer drugs interfering with prostaglandin and leukotriene biosynthesis.^2^ Contrary to the classical non-steroidal anti-inflammatory drugs (NSAIDs), namely blockers of cyclooxygenases (COX-1 and COX-2) and coxibs (COX-2 selective inhibitors), the inhibition of mPGES-1 does not affect the biosynthesis of the other physiologically important PGs.^3,4^ Consequently, mPGES-1 inhibitors show a safer profile with respect to fewer gastrointestinal and cardiovascular complications, like thrombosis and vascular inflammation.^5,6^ Several studies reported the involvement of this synthase in different types of cancer,^7−9^ liver diseases, like viral hepatitis, and drug-induced injury.^10^ To date, only two drug candidates are currently in Phase II clinical trials: GRC 27864 is being evaluated for efficacy in patients with osteoarthritic pain; GS-248 is currently being tested in a Phase II trial (https://clinicaltrials.gov/ct2/show/NCT04744207) in Europe with systemic sclerosis patients (https://clinicaltrials.gov/ct2/results?term=mPGES-1). Thus, the development of mPGES-1^1,10,11^ inhibitors represents an urgent issue. Furthermore, in recent years, different series of dual- and/or multi-target inhibitors of eicosanoid biosynthesis targets have been developed. In fact, the use of this type of agents able to block the targets belonging to the three different branches of the arachidonic acid cascade, namely lipoxygenases (LOs), cyclooxygenases (COXs) and cytochrome P_450_ monooxygenases (CYP450), may increase the anti-inflammatory effects and reduce the side effects. Indeed, the moderate interference with multiple biological macromolecules may provide advantages in re-adjusting and regulating homeostasis compared to single-target drugs, obtaining the next generation of more efficient and safer anti-inflammatory agents.^12^ In the continuous effort to identify mPGES-1 inhibitors, computational tools have always played a central role.^13−15^ In this context, considering the broad spectrum of biological activities^16,17^ of the 2-amino-thiadiazole derivatives, such as antifungal^18^ and antiparasitic activities,^19^ and also encouraged by the inhibitory activity shown by 2-aminothiazole-based mPGES-1 inhibitors,^20,21^ we investigated the privileged scaffold 2-aminoacyl-1,3,4-thiadiazole as central core for designing potential mPGES-1-blocking agents. To target mPGES-1 protein, in these past few years, we improved and optimized a multi-step computational workflow integrated with robust *in vitro*, *in vivo*, and *ex vivo* experimental analyses^22,23^ that allowed us to identify novel dual mPGES-1 and leukotriene biosynthesis inhibitors. Therefore, the generation of a novel library of compounds was the first step in starting our investigation by identifying promising specific chemical platforms for a punctual decoration to be performed according to a selected synthetic approach. Thus, according to the generic scheme reported in Figure 1A, the 2-amino-5-(4-bromophenyl)-1,3,4-thiadiazole scaffold was decorated with commercially available acyl chlorides (i.e., 318) and boronic acids (i.e., 570) (Combiglide, LigPrep, QikProp software, see Supporting Information).^24^ Considering our final purpose of the *in vivo* evaluation of the most promising hits, during our investigation we used several computational facilities (Schrödinger Suite 2021)^24^ to obtain a small pool of molecules endowed with both favorable pharmacokinetic properties and encouraging pharmacodynamic effects. For these reasons, we first used the LigPrep module to generate all the possible tautomers and protonation states at pH = 7.4 for all molecules belonging to the 2-amino-thiadiazole-based library, obtaining ∼4.2 × 10^5^ entities. After that, QikProp and LigFilter software was used to filter out only compounds presenting the well-known “drug-like” properties. To discard “non-drug-like” compounds and possible false positives in high-throughput screening (HTS) assays, QikProp software^24^ was used for the calculation of the pharmacokinetic properties, physically significant descriptors, and pharmaceutically relevant parameters for prediction of absorption, distribution, metabolism, and excretion (ADME). Accordingly, the functional groups generally responsible for reactivity, toxicity, or decomposition problems *in vivo* were filtered out before the subsequent molecular docking step, in order to rule out “non-drug-like” molecules (Table S1, Supporting Information). Then, the virtual screening workflow (VSW) on mPGES-1 (PDB code: 4BPM)^25^ was applied to the final library, containing 1.5 × 10^5^ compounds that passed several filters (*vide supra*), using Glide software.^24^ Specifically, the VSW consisted of three subsequent steps, each of them yielding a ranking of compounds according to docking score value: (i) high-throughput virtual screening phase (HTVS); (ii) standard precision phase (SP); and (iii) extra precision phase (XP). The computational analyses of docking results were performed by combining the docking score with a qualitative and visual inspection of a specific set of ligand/mPGES-1 interactions responsible for the inhibitory activity, as already reported by other research groups and by us.^22,26−28^ In more detail, considering that a known mPGES-1 inhibitor is able to occupy the peculiar binding groove with a U-shape of the ligand-binding site, in our *in silico* evaluation the interactions with specific aromatic (i.e., Tyr28_ChainC_, Phe44_ChainC_, Tyr130_ChainA_), aliphatic (i.e., Val24_ChainC_, Val128_ChainA_, Leu132_ChainA_), polar (i.e., Pro124_ChainA_, Ser127_ChainA_, Thr131_ChainA_), and charged (i.e., Arg52_ChainC_ and Gln134_ChainA_) residues of the pharmacological site of interest were considered for the selection of the best chemical candidates. Specifically, for all the predicted most favored docking poses, the substituents deriving from the side chain of the acyl chlorides preferentially interact with key amino acids, namely Phe44_chainC_, His53_chainC_, Gly35_ChainC_, Asp49_ChainA_, and Ser127_ChainA_ of the cytoplasmic part of the protein (Figure S1, Supporting Information), while the side chains related to the substitution with the boronic acids (Figure 1A) establish interactions with the binding pocket (key residues: Val24 _ChainC_, Tyr28 _ChainC_, Tyr130 _ChainA_, Gln134 _ChainA_, Val 128 _ChainA_, Thr131 _ChainA_, and Leu132 _ChainA_). Finally, our scientific workflow led to selecting the most promising hits by applying a further filter for excluding the “Pan-Assay Interference compounds” using the SwissADME web tool.^29^ This computational tool has allowed a further accurate selection of the best candidates (compounds **1**–**9**, Figure 1B) for (i) chemical synthesis; (ii) biological evaluation in both cell-free and cell-based systems; and (iii) *in vivo* and *ex vivo* investigation of the anti-inflammatory properties of the most promising hits.

**Figure 1:**
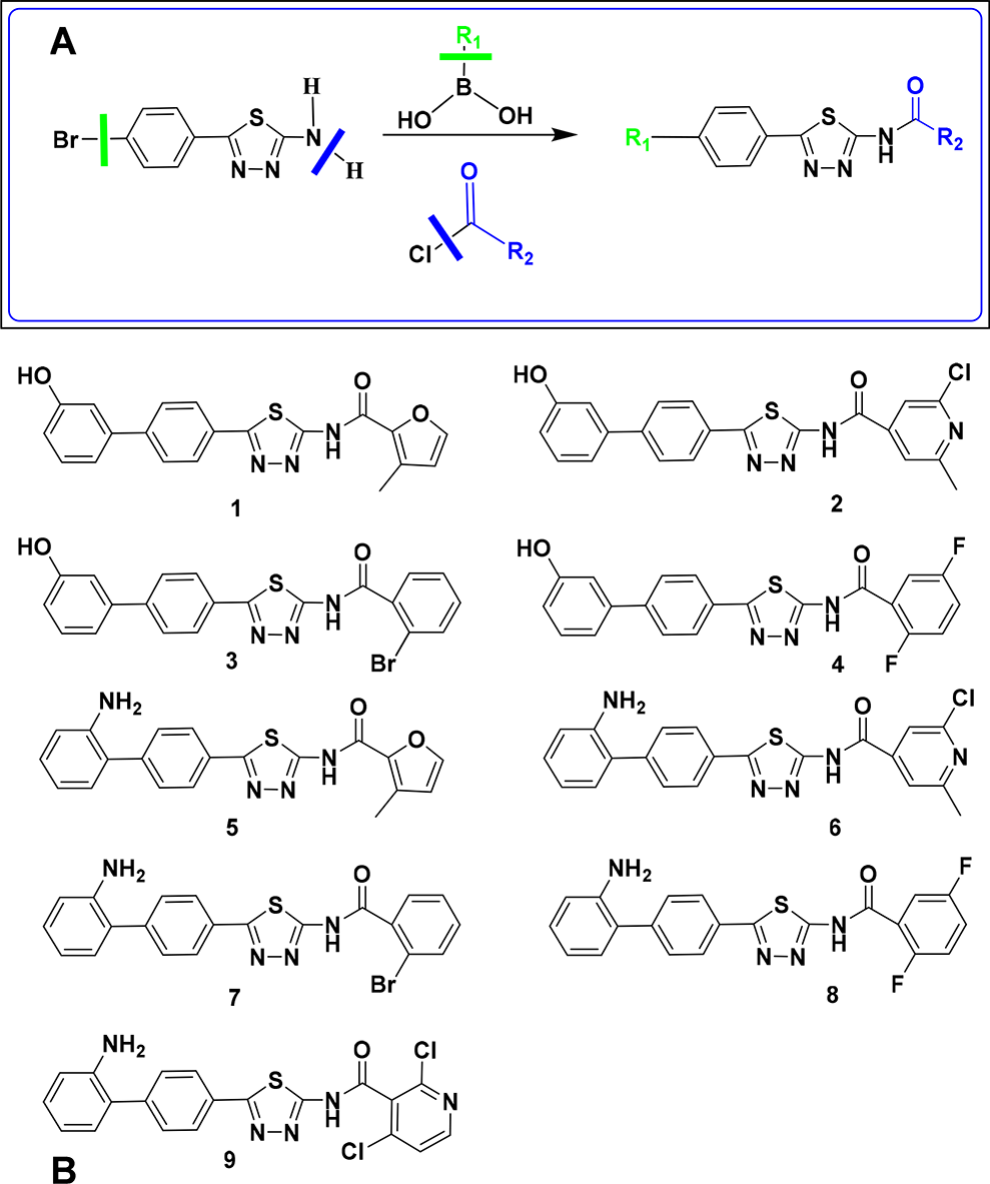
(A) Generation of a library of *N*-(5-(4-arylphenyl)-1,3,4-thiadiazol-2-yl)arylcarboxamides starting from 2-amino-1,3,4-thiadiazole. (B) Chemical structures of compounds **1–9** deriving from the virtual screening workflow focused on structure-based docking analysis.

The novel set of selected compounds targeting mPGES-1 was then synthesized according to the synthetic route reported in Scheme 1. Compounds **1**–**9** can be divided into groups that differ in some chemical features: (i) compounds bearing the 3-hydroxyphenyl moiety (**1**–**4**) and (ii) compounds bearing the 2-aminophenyl moiety (**5**–**9**). In both cases, the general synthetic route consisted of two main steps: 2-amino-5-(4-bromophenyl)-1,3,4-thiadiazole was subjected to Pd-catalyzed Suzuki–Miyaura^30^ cross-coupling with 3-hydroxyphenylboronic acid pinacol ester **I** (line 1, Scheme 1) or 2-(*N*-Boc-amino)phenylboronic acid **II** (line 2, Scheme 1) to give **10** and **11**, respectively. For the synthesis of these compounds, the Suzuki–Miyaura reaction was performed under standard conditions using [1,1′-bis(diphenylphosphino)ferrocene]dichloropalladium(II) as the catalyst and aqueous carbonate as base. The intermediates **10** and **11** were then subjected to an acylation reaction on the amino group of the 2-aminothiadiazole ring. Due to the presence of the reactive phenolic group, compound **10** was protected by reaction with trimethylsilyl chloride, and then the proper acyl chlorides were added *in situ*.

**Scheme 1:**
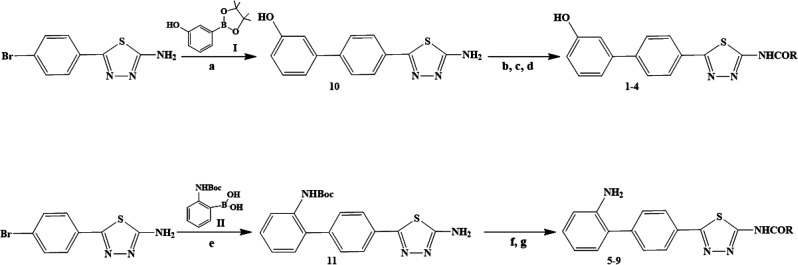
Synthetic Chemical Routes Used for the Syntheses of Compounds **1**–**11** Line 1, reagents and conditions: (a) **I**, K_2_CO_3_, Pd(dppf)Cl_2_, 1,4-dioxane–H_2_O, 80 °C; (b) (CH_3_)_3_SiCl, pyridine, CH_3_CN, r.t.; (c) RCOCl; (d) HCl. Line 2, reagents and conditions: (e) **II**, K_2_CO_3_, Pd(dppf)Cl_2_, 1,4-dioxane–H_2_O; (f) RCOCl, pyridine, CH_3_CN, r.t.; (g) TFA, DCM, r.t.

The protecting group was then removed by means of an acid workup of the reaction mixture in order to obtain the final compounds. For compounds **5**–**9**, the *tert*-butoxycarbonylamino (Boc)-protected boronic acid **II** was used in the coupling step. The product of the Suzuki reaction was then subjected to acylation with the appropriate acyl chloride, and then the Boc group was removed with a mixture of DCM (50%) and TFA (50%) to give the deprotected aminophenyl derivatives.

To corroborate our computational outcomes, the synthesized compounds were screened for inhibition of PGE_2_ biosynthesis at 10 μM using a cell-free assay^31^ (Table 1). Compounds **3**, **6**, **7**, and **9** represented the most promising inhibitors, which caused high inhibitory activity with IC_50_ = 0.2 ± 0.0 μM, 3.1 ± 0.6 μM, 0.15 ± 0.0 μM, and 1.7 ± 0.3 μM, respectively (Table 1 and Figure S2, Supporting Information). Interestingly, like our previous study on aminobenzothiazole derivatives,^13^ the 2-bromophenylcarbonyl moiety in compounds **3** and **7** is confirmed to be capable of establishing robust interactions with mPGES-1. From the structural point of view, the biological affinities of compounds **3** and **7** (Figure 2A and C) could be positively affected by the 2-bromobenzoyl substituent able to accommodate in a deep binding pocket on the mPGES-1 surface. This moiety interacts by van der Waals contacts with amino acids of the cytoplasmic part and establishes π-stacking with Phe44_ChainC_ and His53_ChainC_ hydrogen bonds with Ser127, and a halogen bond between the bromine atom and the side chain of Aps49_ChainC_. Furthermore, the 3-phenylphenol and 2-aminobiphenyl moieties of **3** and **7** (Figure 2A and C), respectively, are able to establish a peculiar π-stacking with Tyr130_ChainA_ and a hydrogen bond with Gln134_ChainA_. A comparable binding mode is also observed for **6** and **9** (Figure 2B and D), where also in these cases, the presence of halogen could represent a critical factor in affecting the mPGES-1 activity.

**Table 1:** Residual PGE_2_ Biosynthesis of Compounds **1**–**9** in the Cell-Free Assay, Expressed as Percentage of Control (100%)

compound	mPGES-1 residual activity at 10 μM (%)	IC_50_ value ± SEM (μM)^a^
**1**	>50	n.d.
**2**	>50	n.d.
**3**	8.4	0.2 ± 0.0
**4**	>50	n.d.
**5**	>25	n.d.
**6**	31.7	3.1 ± 0.6
**7**	16.7	0.15 ± 0.0
**8**	>50	n.d.
**9**	20.9	1.7 ± 0.3

a *n* = 3; n.d., not determined.

**Figure 2:**
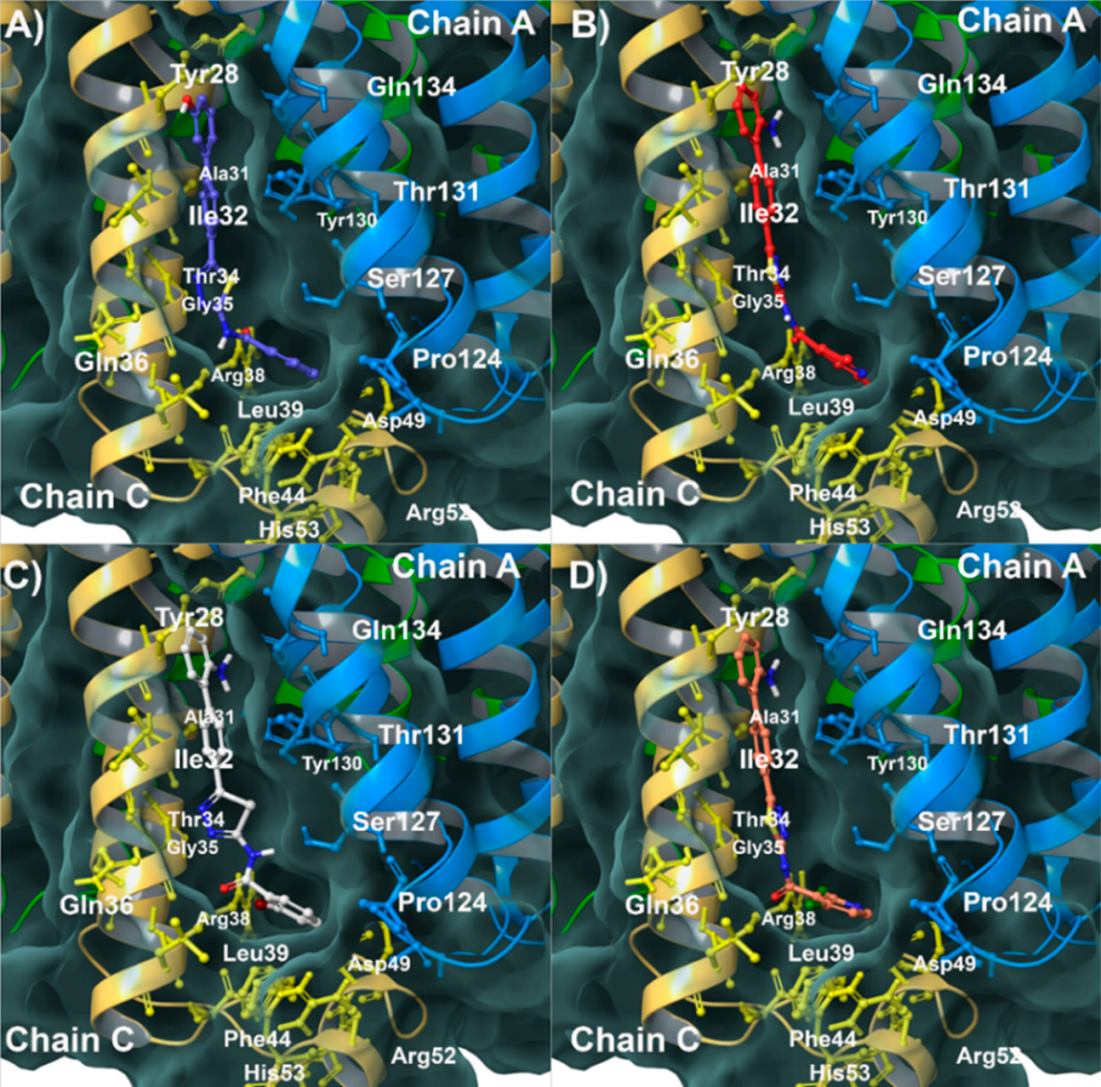
(A) **3** (colored by atom type: C violet, O red, N blue, polar H light gray), (B) **6** (colored by atom type: C red, O red, N blue, polar H light gray), (C) **7** (colored by atom type: C white, O red, N blue, polar H light gray), and (D) **9** (colored by atom type: C salmon pink, O red, N blue, polar H light gray) in complex with mPGES-1. The transparent molecular surface is colored in black, and the secondary structure and key residues are reported as ribbons and sticks colored by chain (i. e., Chain A in light blue and Chain C in yellow).

Since compounds **3**, **6**, **7**, and **9** displayed the most potent effects, cell-free assays were performed on several related enzymes involved in the inflammatory response to deeply investigate their anti-inflammatory features and to evaluate their selectivity versus mPGES-1. None of the investigated compounds was active against isolated cyclooxygenases (COX)-1/2, 5-lipoxygenase (5-LO), and soluble epoxide hydrolase (sEH) (Table 2). Additionally, the effect of compounds **3**, **6**, **7**, and **9** on PGE_2_ production was evaluated in a cell-based system, namely in IL-1β-stimulated A549 cells. Compounds **6**, **7**, and **9** were able to suppress the biosynthesis of PGE_2_ in a concentration-dependent manner (Figure 3A).

**Table 2:** Residual Activity of Isolated Enzymes Involved in Inflammatory Eicosanoid Formation in the Presence of Compounds **3**, **6**, **7**, and **9**

	residual activity (%)^a^			
compound	COX-1	COX-2	5-LO	sEH
**3**	n.i. (104)	n.i. (65.4)	n.i. (125)	n.i. (96)
**6**	n.i. (82.3)	n.i. (83.1)	42.8 ± 1.6^b^	n.i. (67.7)
**7**	n.i. (98.1)	n.i. (194)	24.3 ± 1.1^b^	n.i. (164)
**9**	n.i. (107)	n.i. (72.7)	n.i. (75.9)	n.i. (83.9)

a Data are expressed as percentage of uninhibited control (100%), *n* = 3. n.i.: no inhibitory activity observed.

b IC_50_ value ± SEM (μM).

**Figure 3:**
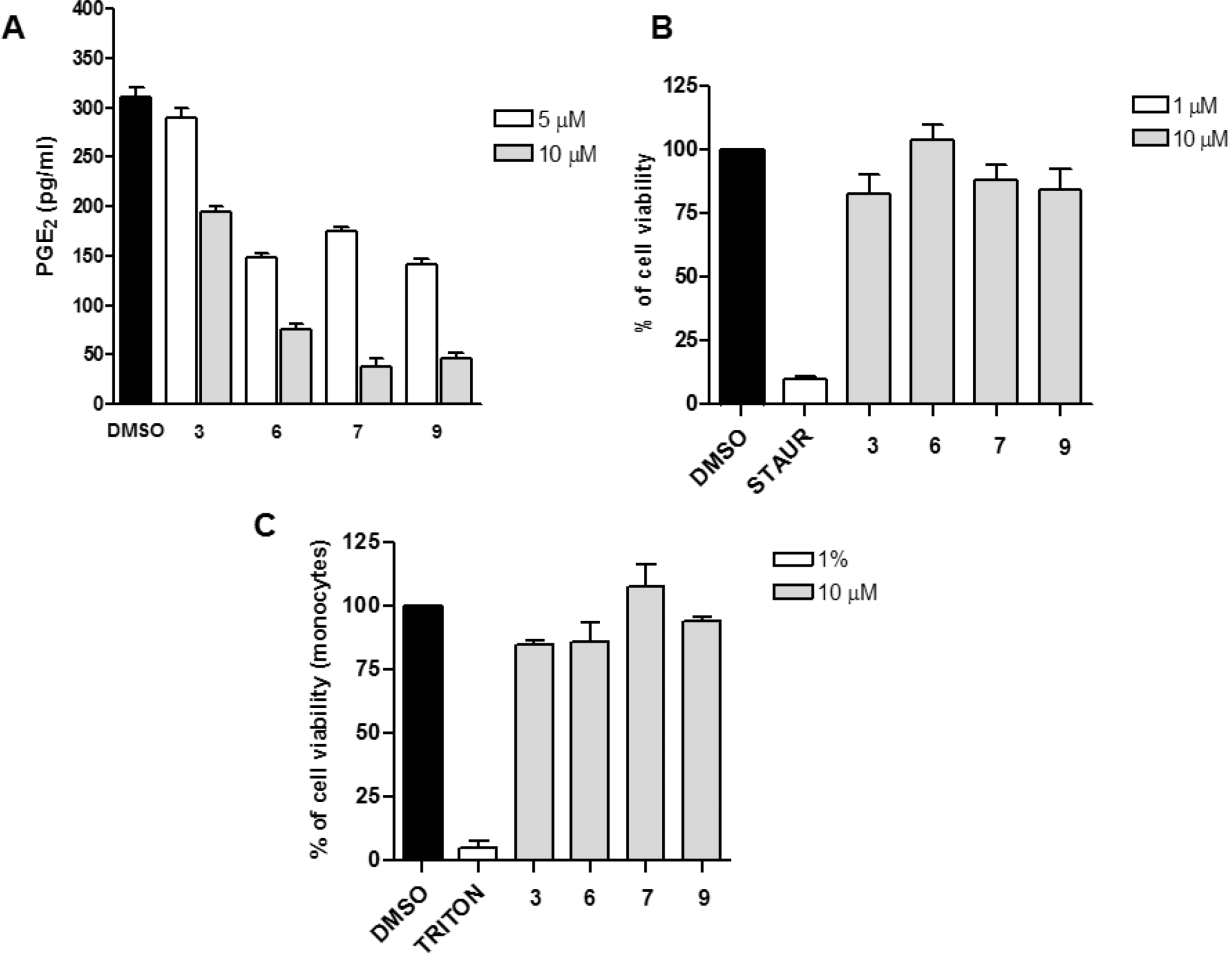
(A) A549 cells in conditioned medium (1% FBS and 10 ng/mL IL-1β) were incubated for 24 h with 5 and 10 μM of compound **3**, **6**, **7**, and **9**. PGE_2_ released into the medium was quantified using a specific ELISA. Control cells were stimulated with IL-1β, treated with vehicle (DMSO), and expressed as mean ± SEM (pg/mL), *n* = 2. (B) A549 cell line was used to perform a MTT assay. A549 were treated with **3**, **6**, **7**, and **9** (test compounds, 10 μM), staurosporine (1 μM, positive control), or vehicle (0.1% DMSO) for 48 h. (C) Human monocytes were used to perform a MTT assay. Cells were treated with **3**, **6**, **7**, and **9** (test compounds, 10 μM), Triton X-100 (1%, positive control), or vehicle (0.5% DMSO) for 24 h. Data are expressed as a percentage of control (100%), means, SEM, *n* = 3.

Moreover, cell viability assays performed on the A549 cell line and human monocytes excluded that the effects on the levels of PGE_2_ were related to possible cytotoxicity of the tested compounds (Figure 3B, C). Furthermore, the interference of compounds **3**, **6**, **7**, and **9** on the leukotriene biosynthesis was investigated in cell-based systems. Two different experimental settings were applied using intact neutrophils from human peripheral blood: stimulation with Ca^2+^-ionophore or with Ca^2+^-ionophore plus arachidonic acid as exogenous substrate. Interestingly, compounds **3**, **6**, **7**, and **9** inhibited the formation of LTB_4_, its isomers, and 5-H(p)ETE, presenting promising IC_50_ values (Table 3 and Figure S3, Supporting Information).

**Table 3:** 5-LO Product Formation in Intact Neutrophils after Incubation with Compounds **3**, **6**, **7**, and **9** Stimulated with Either A23187 or A23187 plus 20 μM Arachidonic Acid (AA)

	5-LO product formation on PMNL^a^	
compound	*stimulus*: A23187	*stimulus*: A23187 + AA
**3**	4.9 ± 0.5	5.2 ± 1.2
**6**	4.8 ± 1.9	6.9 ± 2.2
**7**	4.2 ± 1.4	2.3 ± 0.8
**9**	2.1 ± 0.3	4.2 ± 0.3

a IC_50_ value ± SEM (μM), *n* = 3.

The absence of 5-LO inhibition in the cell-free assay (Table 2), in fact, does not exclude an inhibitory activity on FLAP, which is operative in intact cells, and its inhibition by test compounds may explain the impaired cellular LT formation. We confirmed potent inhibition of cellular 5-LO product formation also in human pro-inflammatory macrophages activated by *Staphylococcus aureus* for 90 min by **3**, **6**, **7**, and **9** but not by **2** (used as negative control), as expected (Figure S4, Supporting Information). Notably, the formation of other products derived from 12/15-LOXs was not suppressed but rather elevated. Successively, we sought to investigate the potential inhibitory activity of tested compounds on J774A.1 macrophage stimulated with LPS (10 μg/mL). As shown in Figure 4, compound **3** (1 μM) was able to modulate both IL-6 (Figure 4A) and PGE_2_ (Figure 4B) production (*P* ≤ 0.01) in macrophages stimulated with LPS. Compound **7**, at the same concentration, displayed a similar activity only in terms of PGE_2_ modulation (*P* ≤ 0.05). The *in vitro* biological activity assays disclosed compound **3** as the most promising anti-inflammatory agent. Therefore, it was selected to evaluate the leukocytes egress into the peritoneal cavity in a mouse model of zymosan-induced peritonitis (see Supporting Information).^32−34^

**Figure 4:**
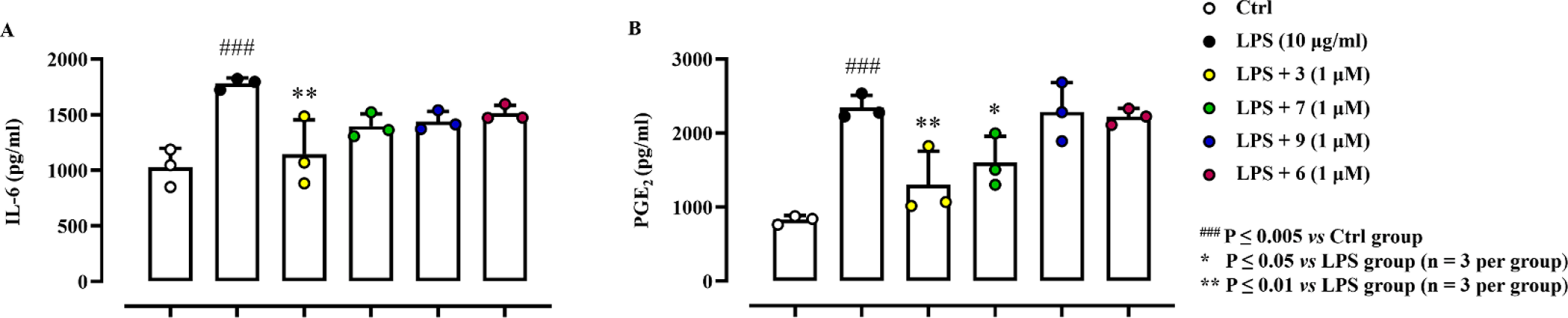
Effects of test compounds on the release of classical pro-inflammatory mediators such as (A) IL-6 and (B) PGE_2_ from murine macrophages (J774A.1) post LPS stimulation. Results (pg/mL) are expressed as mean ± SD. For statistical analysis, see SI.

Mice were subjected to intraperitoneal (i.p.) injection of 500 mg/kg zymosan, followed by injection of compound **3**. Intraperitoneal injections of PBS alone and of dexamethasone (3 mg/kg) 30 min after zymosan administration were also carried out as an internal control. The strong leucocyte recruitment due to zymosan injection was reduced by administration of compound **3** at the dose of 10 mg/kg at both 4 (Figure 5A; *P* ≤ 0.05) and 24 h (Figure 5B; *P* ≤ 0.01).

**Figure 5:**
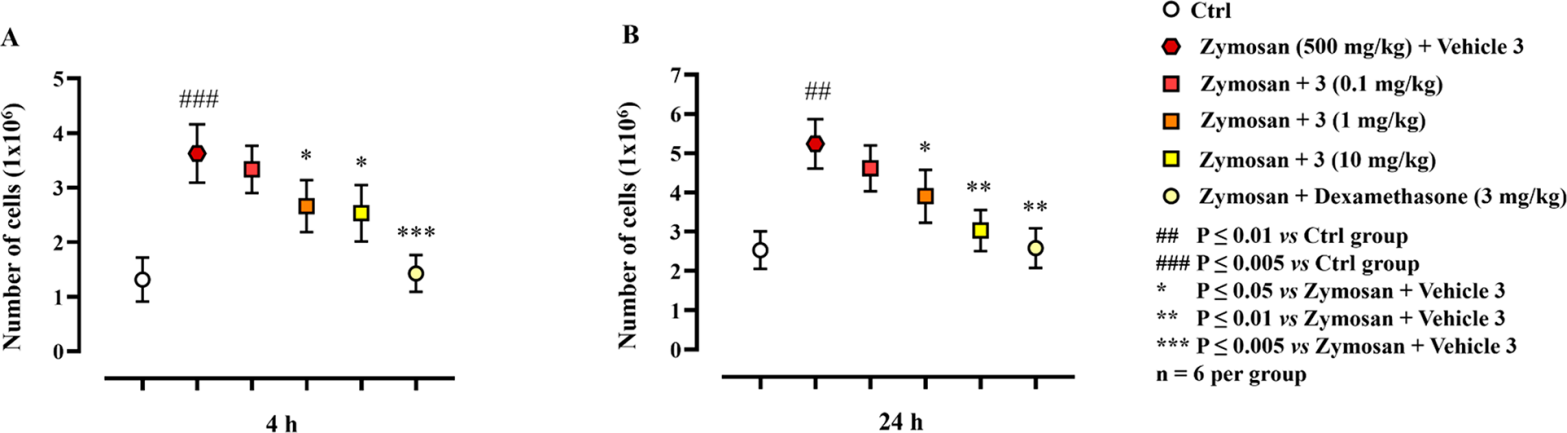
Effect of compound **3** in zymosan-induced peritonitis in mice. Mice were randomly divided into different experimental groups: control group (Ctrl), model group (zymosan + vehicle compound **3**), zymosan + compound **3** (i.p. injection 30 min after i.p. injection of zymosan) group, and zymosan + dexamethasone (i.p. injection 30 min after i.p. injection of zymosan) group. At (A) 4 and (B) 24 h after injection, peritoneal exudate from each mouse was recovered, and the total cell number was evaluated. Results are expressed as mean ± SD. For statistical analysis see SI.

Interestingly, compound **3** showed a remarkable effect even at the lower dose of 1 mg/kg (*P* ≤ 0.05) at both time points (Figure 5A, B). Furthermore, leukocyte numbers in the peritoneal cavity at both 4 (*P* ≤ 0.005; Figure 5A) and 24 h (*P* ≤ 0.01; Figure 5B) were significantly reduced by dexamethasone. A single administration of zymosan (500 mg/kg) at 4 and 24 h induced a substantial increase in the levels of IL-1β (Figure 6A, B), IL-6 (Figure 6C, D), and PGE_2_ (Figure 6E, F) compared to control group. Conversely, a significant reduction of IL-10 levels was observed at both time points (Figure 6G, H). In addition, IL-1β (Figure 6A, B; *P* ≤ 0.05 and *P* ≤ 0.01 at 4 and 24 h, respectively), IL-6 (Figure 6C, D; *P* ≤ 0.05 and *P* ≤ 0.01 at 4 and 24 h, respectively), and PGE_2_ (Figure 6E, F; *P* ≤ 0.01 at both 4 and 24 h) levels were significantly reduced by compound **3** at 10 mg/kg. The IL-10 level was modulated only at 24 h (Figure 6H; *P* ≤ 0.05). Injection of dexamethasone (3 mg/kg) modulated the values of IL-1β, IL-6 PGE_2_, and, conversely, IL-10 with a more prominent effect (Figure 6).

**Figure 6:**
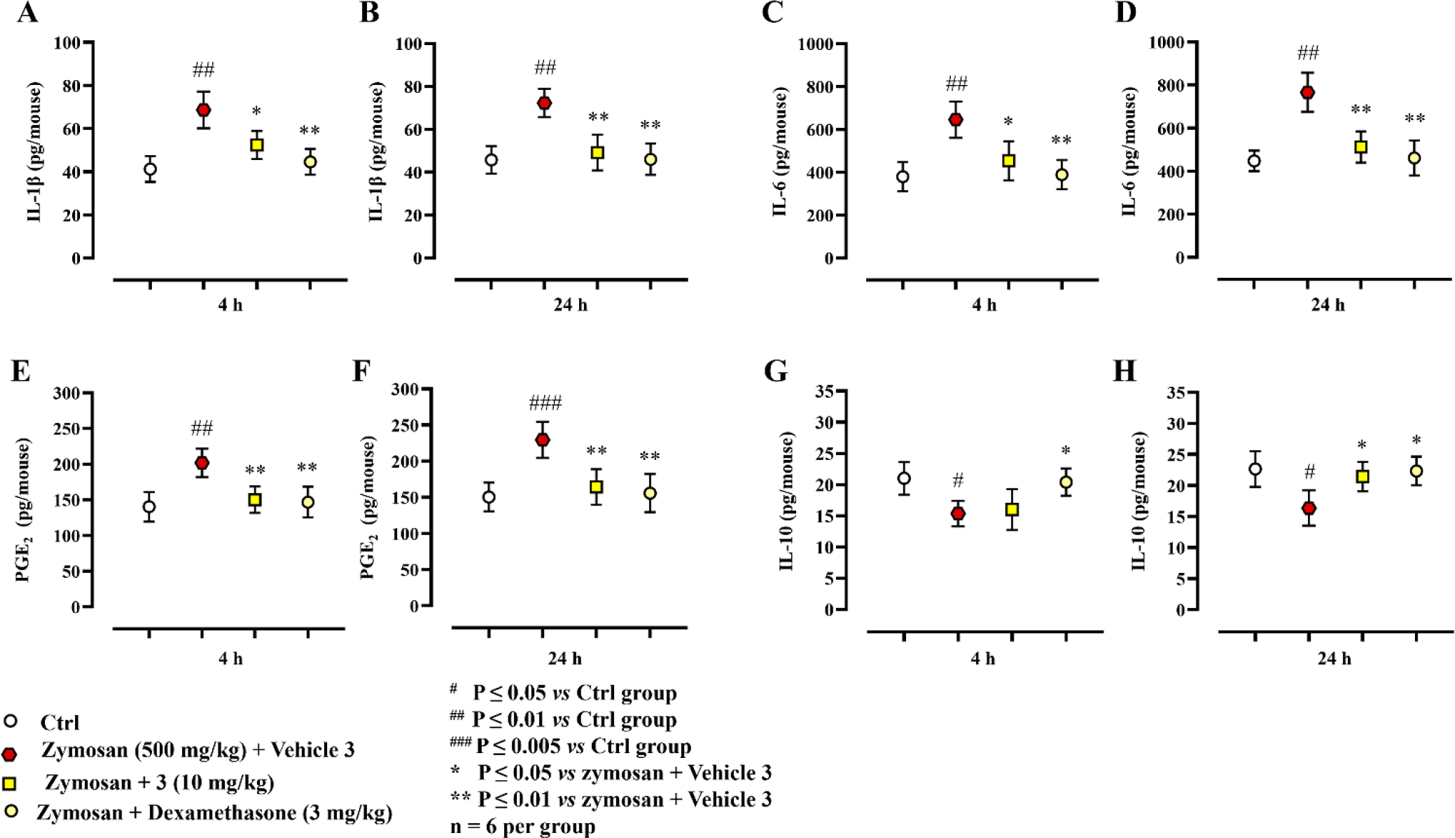
Analysis of cytokine levels collected in the peritoneal exudate of mice from different experimental groups: (A, B) IL-1β, (C, D) IL-6, (E, F) PGE_2_, and (G, H) IL-10. Results (normalized to exudate levels) are expressed as mean ± SD. For statistical analysis see SI.

In order to overcome the well-known issue related to the inefficacy of some selective human mPGES-1 inhibitors in mice due to the structural differences in target proteins (i.e., Arg52, which is Lys53 in murine mPGES-1, and His53, which is Arg54 in murine mPGES-1),^35^ we also performed homology modeling and molecular docking studies on a murine mPGES-1 model (see Supporting Information).

Specifically, we disclosed the putative interactions of our hits **3**, **6**, **7**, and **9** with the highly conserved region of the active site of murine mPGES-1, ensuring that the 2-amino-1,3,4-thiadiazole-based compounds could similarly bind both the isoforms, showing comparable binding modes in the active sites of the enzymes (see Supporting Information and Figure S6). In line with the widespread presence of thiadiazole in the chemical structure of several pharmacologically active compounds, we here demonstrated that 2-amino-1,3,4-thiadiazole-based compounds, when opportunely decorated, are promising anti-inflammatory hits. The application of a multi-step computational protocol, based on a concise synthetic method, allowed us to single out diverse substituted 2-acylamino thiadiazole derivatives, which led to the identification of the novel hits **3**, **6**, **7**, and **9** as potent PGE_2_ biosynthesis inhibitors, with **3** and **7** showing the strongest effects. In addition, the compounds were screened against several enzymes involved in the inflammatory response using cell-free assays. First, no activity was found against COX-2 as well as the constitutively expressed COX-1, ensuring the absence of the well-known side effects due to the action on COX targets (*vide supra*), making these compounds interesting candidates as safer alternatives, especially for long-term therapies.

Moreover, the compounds were not able to interfere with 5-LO activity in cell-free assays, while their ability to strongly interfere with cellular biosynthesis of leukotrienes was demonstrated, presumably due to interference with FLAP. Finally, *in vivo* and *ex vivo* results also demonstrated that the zymosan-induced leukocyte migration was attenuated by treatment with compound **3**, with a significant modulation in the levels of typical pro-/anti-inflammatory cytokines with efficacy similar to that of dexamethasone. Finally, we performed homology modeling and molecular docking toward the murine mPGES-1 to corroborate the potential utility of this molecular scaffold as a modulator of the PGE_2_ level in both murine and human models.

In summary, our multidisciplinary workflow, which combines *in silico* studies, chemical synthesis, and cell-free and cell-based assays, represents a fast and powerful method for identifying novel hits that are able to inhibit PGE_2_ and LT biosynthesis with *in vivo* anti-inflammatory activity.

## Supporting Information Available

Experimental details regarding computational, synthetic, *in vitro*, *in vivo*, and *ex vivo* procedures; additional figures illustrating 3D and 2D interactions, NMR spectra, HRMS spectra, HPLC traces (PDF)

## Abbreviations

DREAM-in-CDM: Drug Repurposing Effort Applying Integrated Modeling-in vitro/in vivo-Clinical Data Mining
5S-HETE: 5*S*-hydroxy-eicosatetraenoic acid
ELISA: enzyme-linked immunosorbent assay
FBS: fetal bovine serum
FLAP: 5-lipoxygenase-activating protein
HTS: high-throughput screening
HTVS: high-throughput virtual screening
IL: interleukin
LO: lipoxygenase
5-LOX: 5-lipoxygenase
LPS: lipopolysaccharide
LTB4: leukotriene B4
LTC4S: leukotriene C4 synthase
MAPEG: metabolism of glutathione and prostanoids
mPGES-1: microsomal prostaglandin E_2_ synthase-1
MTT: 3-(4,5-dimethylthiazol-2-yl)-2,5-diphenyltetrazolium bromide
NSAID: non-steroidal anti-inflammatory drug
PG: prostaglandin
PGE_2_: prostaglandin E_2_
PGH_2_: prostaglandin H_2_
PMNL: polymorphonuclear leukocyte
sEH: soluble epoxide hydrolase
SP: standard precision
VSW: virtual screening workflow
XP: extra precision
