# Supplementary material for: Identification of 2-Aminoacyl-1,3,4-thiadiazoles as Prostaglandin E2 and Leukotriene Biosynthesis Inhibitors
Source: ACS Med Chem Lett. 2022 Dec 9;14(1):26–34. doi: 10.1021/acsmedchemlett.2c00343 (PMC9841589; doi:10.1021/acsmedchemlett.2c00343)
Supplement: Supplementary file 1 — ml2c00343_si_001.pdf [file ml2c00343_si_001.pdf]

Identification of 2-aminoacyl-1,3,4-thiadiazoles as prostaglandin E_2 and leukotriene biosynthesis inhibitors

Marianna Potenza,[a, b, ‡] Assunta Giordano,[a, c, ‡] Maria G. Chini,[d] Anella Saviano,[e] Christian Kretzer,[f] Federica Raucci,[e] Marina Russo,[e] Gianluigi Lauro,[a] Stefania Terracciano,[a] Ines Bruno,[a] Maria Iorizzi,[d] Robert K. Hofstetter,[f] Simona Pace,[f] Francesco Maione,[e] Oliver Werz,[*, f] Giuseppe Bifulco[*, a]

[a]*Department of Pharmacy, University of Salerno, via Giovanni Paolo II, 132, 84084, Fisciano, Italy.*
[b]*The FIRC Institute of Molecular Oncology, Via Adamello 16, 20139 Milan, Italy.*
[c]*Institute of Biomolecular Chemistry (ICB), Consiglio Nazionale delle Ricerche (CNR), Via Campi Flegrei 34, I-80078, Pozzuoli, Napoli, Italy.*
[d]*Department of Biosciences and Territory, University of Molise, Contrada Fonte Lappone, Pesche, Isernia, I-86090, Italy.*
[e]*ImmunoPharmaLab, Department of Pharmacy, School of Medicine and Surgery, University of Naples Federico II, Via Domenico Montesano 49, 80131 Naples, Italy.*
[f]*Department of Pharmaceutical/Medicinal Chemistry, Institute of Pharmacy, Friedrich Schiller University Jena, Philosophenweg 14, 07743 Jena, Germany.*

[‡]The authors contributed equally to this work

Table of Contents

Table S1: Calculated properties and descriptors according to QikProp software of synthetized compounds 1-9. 5

Table S1: (continued) 6

Figure S1. Representative interaction mode of the most promising 1,3,4-thiadiazole-based scaffold molecules in the mPGES-1 receptor counterpart (pdb code: 4BPM, colored by chain). 7

Figure S2. Concentration-response curves of compounds 3, 6, 7, 9 for inhibition of mPGES-1 in a cell-free assay. IC50 values = 0.2 ± 0.0 µM, 3.1 ± 0.6 µM, 0.15 ± 0.0 µM and 1.7 ± 0.3 µM, respectively. Data are expressed as percentage of control (100%), means ± S.E.M., n = 3. 8

Figure S3. Concentration-response curves for inhibition of 5-LO product formation in neutrophils. 9

Figure S4. Human monocyte-derived macrophages were pre-treated with test compounds (10 µM) or vehicle 15 min prior stimulation 10

Computational procedures 11

Figure S5. The Ramachandran plot showing the phi-psi torsion angles for all residues (except those at the chain termini) for the protein model murine mPGES-1 built by homology modeling.. 13

Figure S6. A) 3 (colored by atom type: C violet, O red, N blue, polar H light grey), B) 6 (colored by atom type: C red, O red, N blue, polar H light grey); C) 7 (colored by atom type: C white, O red, N blue, polar H light grey); D) 9 (colored by atom type: C salmon pink, O red, N blue, polar H light grey) in comlex with model of murine mPGES-1. 15

General synthetic procedures 16

Cell-free mPGES-1 activity assay 21

Cell-free 5-LO activity assay 22

Cell-free COXs activity assay 22

Expression, purification and activity assay of human recombinant sEH 23

Cells 24

Cell lines 24

Cell viability assay on A549 cell line 25

Cell viability assay on monocytes 25

Determination of 5-LO products in neutrophils 26

Determination of LO products in human macrophages 26

Determination of IL-6 and PGE2 in murine macrophages **27**

Animals **27**

Induction of peritonitis in mice and Enzyme-Linked Immunosorbent Assay (ELISA) **27**

Statistical analysis **28**

Figure S7. ^{1}H NMR (DMSO-d_6, 400 MHz) of compound 1. .... **30**

Figure S8. DEPTQ NMR (DMSO-d_6, 100 MHz) of compound 1 .... **31**

Figure S9. ^{1}H NMR (DMSO-d_6, 400 MHz) of compound 2. .... **32**

Figure S10. DEPTQ NMR (DMSO-d_6, 100 MHz) of compound 2 .... **33**

Figure S11. ^{1}H NMR (DMSO-d_6, 400 MHz) of compound 3. .... **34**

Figure S12. ^{13}C NMR (DMSO-d_6, 100 MHz) of compound 3. .... **35**

Figure S13. ^{1}H NMR (DMSO-d_6, 400 MHz) of compound 4. .... **36**

Figure S14. DEPTQ NMR (DMSO-d_6, 100 MHz) of compound 4 .... **37**

Figure S15. ^{1}H NMR (CD_3OD, 400 MHz) of compound 5 .... **38**

Figure S16. ^{13}C NMR (DMSO-d_6, 100 MHz) of compound 5. .... **39**

Figure S17. ^{1}H NMR (DMSO-d_6, 400 MHz) of compound 6. .... **40**

Figure S18. DEPTQ NMR (DMSO-d_6, 100 MHz) of compound 6 .... **41**

Figure S19. ^{1}H NMR (CD_3OD, 400 MHz) of compound 7 .... **42**

Figure S20. ^{13}C NMR (CD_3OD, 100 MHz) of compound 7 .... **43**

Figure S21. ^{1}H NMR (CD_3OD, 400 MHz) of compound 8 .... **44**

Figure S22. DEPTQ NMR (DMSO-d_6, 100 MHz) of compound 8 .... **45**

Figure S23. ^{1}H NMR (CD_3OD, 400 MHz) of compound 9 .... **46**

Figure S24. DEPTQ NMR (CD_3OD, 100 MHz) of compound 9 .... **47**

Figure S25. HRMS spectrum of compound 3. .... **48**

Figure S26. HRMS spectrum of compound 6. .... **48**

Figure S27. HRMS spectrum of compound 7. .... **49**

Figure S28. HR MS spectrum of compound 9. .... **49**

Figure S29. HPLC chromatogram of compound 3. .... **50**

Figure S30. HPLC chromatogram of compound 6. 50

Figure S31 HPLC chromatogram of compound 7. 51

Figure S32. HPLC chromatogram of compound 9. 51

References 52

Table S1: Calculated properties and descriptors according to QikProp software of synthetized compounds 1-9.

Cmpd	#stars	#rtvFG	mol MW	QPPCaco	WPSA	donorHB	accptHB	QPlogPC16	QPlogPoct	QPlogPw	QPlogPo/w
**1**	0	0	377.4	440.9	39.0	2.0	5.8	13.5	20.3	12.8	3.6
**2**	1	0	422.9	284.3	112.1	2.0	6.3	15.0	22.3	13.3	4.1
**3**	2	0	452.3	459.9	100.0	2.0	5.3	14.9	21.3	12.8	4.5
**4**	1	0	409.4	442.1	122.4	2.0	5.3	13.6	21.1	12.5	4.4
**5**	0	0	376.4	505.3	38.7	2.5	6.0	13.7	21.1	13.8	3.5
**6**	1	0	421.9	328.0	111.5	2.5	6.5	15.2	23.1	14.3	4.0
**7**	2	0	451.3	566.0	105.3	2.5	5.5	15.2	22.1	13.8	4.4
**8**	1	0	408.4	508.3	122.2	2.5	5.5	13.8	21.8	13.6	4.3
**9**	1	0	442.3	371.3	161.5	2.5	6.5	15.4	23.1	14.4	4.2

#stars: Number of property or descriptor values that fall outside the 95% range of similar values for known drugs (**Range or recommended values: 0-5**); **#rtvFG**: number of reactive functional groups, which can lead to false positive in high-throughput screening (HTS) assays and to decomposition, reactivity, or toxicity problem *in vivo* (**Range or recommended values: 0-2**); **#mol_MW**: Molecular weight of the molecule (**Range or recommended values: 130.0 – 725.0**); **#WPSA**: Weakly polar component of the SASA (halogens, P, and S). (**Range or recommended values: 0.0 – 175.0**); **#donorHB**: Estimated number of hydrogen bonds that would be donated by the solute to water molecules in an aqueous solution. Values are averages taken over a number of configurations, so they can be non-integer **(Range or recommended values: 0.0 – 6.0**); #**accptHB**: Estimated number of hydrogen bonds that would be accepted by the solute from water molecules in an aqueous solution. Values are averages taken over a number of configurations, so they can be non-integer **(Range or recommended values 2.0 – 20.0**); **#QPlogPC16:** Predicted hexadecane/gas partition coefficient. (**Range or recommended values 4.0 – 18.0**); **# QPlogPoct:** Predicted octanol/gas partition coefficient. (Range or recommended values 8.0 – 35.0); **#QPlogPw:** Predicted water/gas partition coefficient. (**Range or recommended values 4.0 – 45.0**); **#QPlogPo/w:** Predicted octanol/water partition coefficient. (**Range or recommended values −2.0 – 6.5**);

Table S1: (continued)

Cmpd	QPlogS	QPPCaco	#metab	PercentHumanOralAbsorption	#NandO	RuleOfFive	RuleOfThree
**1**	-5.8	440.9	4	95.3	6	0	1
**2**	-7.0	284.3	3	95.0	6	0	1
**3**	-6.8	459.9	2	100.0	5	0	1
**4**	-6.8	442.1	2	100.0	5	0	1
**5**	-5.7	505.3	5	95.9	6	0	1
**6**	-6.9	328.0	4	95.6	6	0	1
**7**	-6.8	566.0	3	100.0	5	0	1
**8**	-6.7	508.3	3	100.0	5	0	1
**9**	-6.9	371.3	4	100.0	6	0	1

#**QPPCaco:** Predicted apparent Caco-2 cell permeability in nm/sec. Caco-2 cells are a model for the gut-blood barrier. QikProp predictions are for non-active transport. (**Range or recommended values <25 poor, >500 great**); **#metab:** number of likely metabolic reactions (**Range or recommended values: 1 – 8**); **#NandO:** Number of nitrogen and oxygen atoms. (**Range or recommended values 2 – 15**); **#RuleOfFive** (number of violations of Lipinski's rule five, consisting of five rules for drug-like compounds, which require MW < 500, logPo/Pw < 5, number of donor H-bond ≤ 5, number of acceptor H-bond ≤ 10). Compounds that satisfy these rules are considered drug-like. (The "five" refers to the limits, which are multiples of 5) (**Range or recommended values maximum is 4**); RuleOfThree (Number of violations of Jorgensen's rule of three. The three rules are: QPlogS > -5.7, QP PCaco > 22 nm/s, # Primary Metabolites < 7. (**Range or recommended values maximum is 3**).

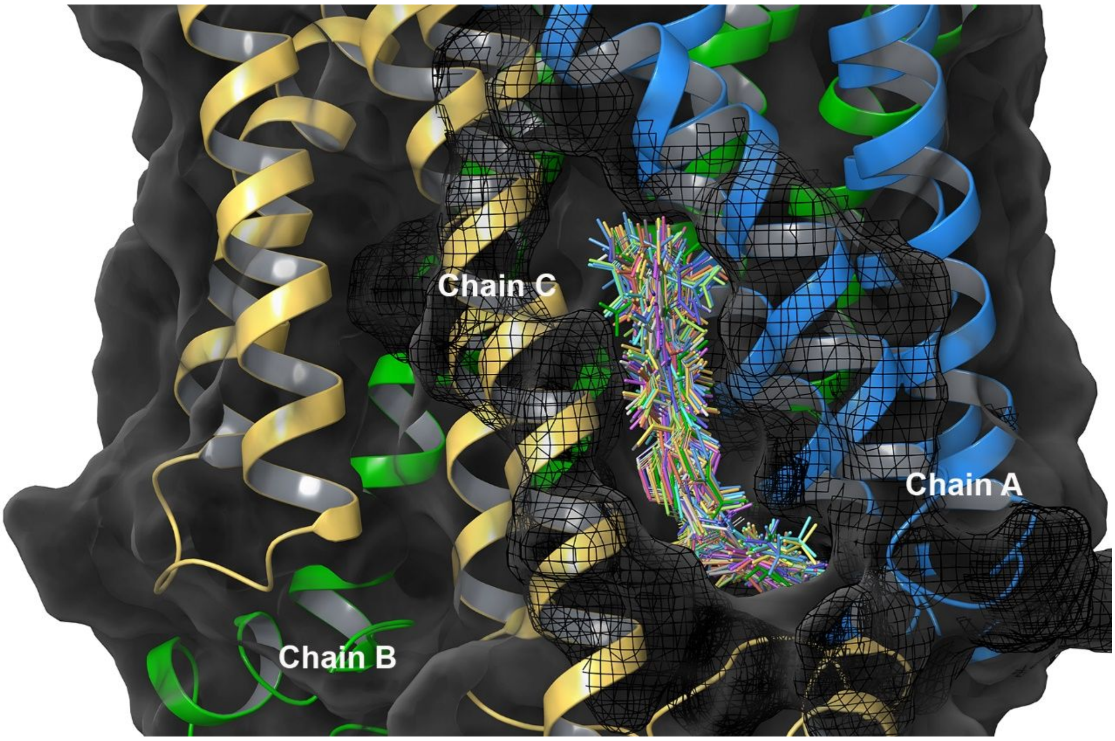

Figure S1. Representative interaction mode of the most promising 1,3,4-thiadiazole-based scaffold molecules in the mPGES-1 receptor counterpart (pdb code: 4BPM, colored by chain).

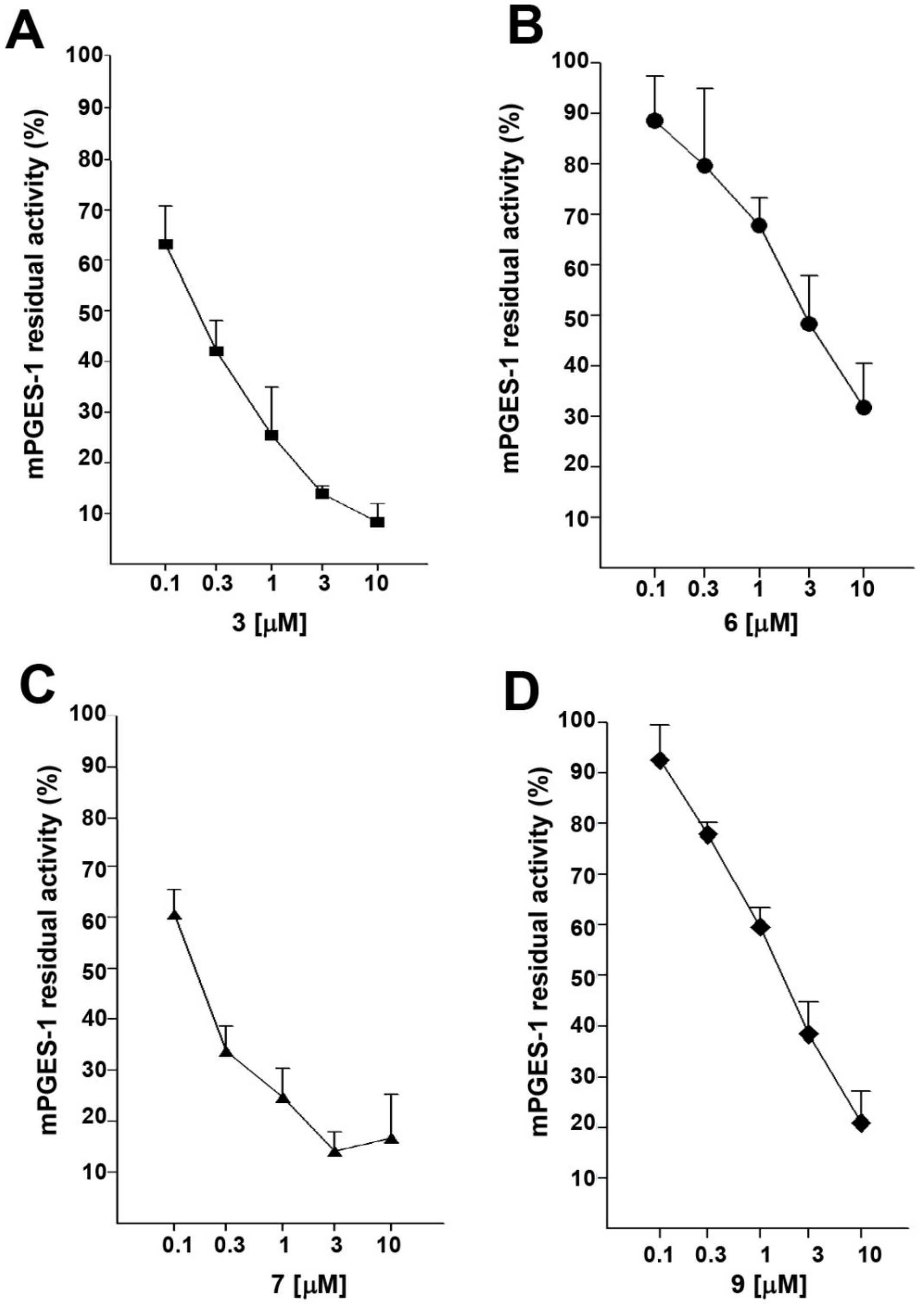

Figure S2. Concentration-response curves of compounds **3**, **6**, **7**, **9** for inhibition of mPGES-1 in a cell-free assay. IC50 values = 0.2 ± 0.0 μM, 3.1 ± 0.6 μM, 0.15 ± 0.0 μM and 1.7 ± 0.3 μM, respectively. Data are expressed as percentage of control (100%), means ± S.E.M., n = 3.

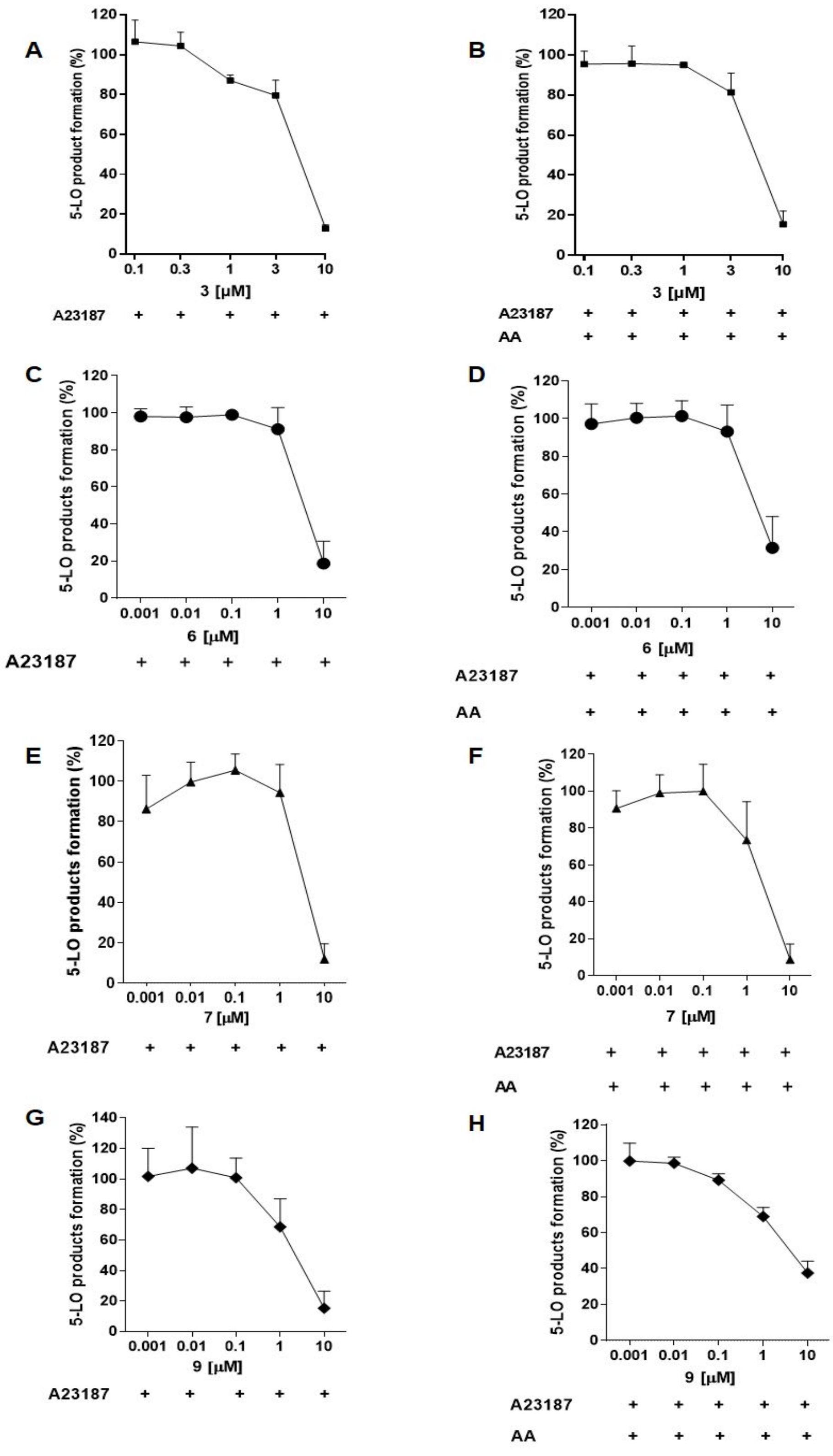

Figure S3. Concentration-response curves for inhibition of 5-LO product formation in neutrophils. Cells were pre-incubated with compounds **3**, **6**, **7** and **9** or 0.1% DMSO over 10 min at 37 °C, and then stimuli were added. On the left (A,C, E, G), cells were stimulated with 2.5 µM Ca^{2+}-ionophore A23187 over 10 min; on the right (B, D, F, H), cells were stimulated with 2.5 µM Ca^{2+}-ionophore A23187 plus 20 µM arachidonic acid over 10 min. Data are expressed as percentage of uninhibited control (100%), means, S.E.M., n = 3.

	3	2	7	9	6
t-LTB_4	8	82	3	3	3
LTB_4	5	93	3	4	4
20-OH-LTB_4	24	83	1	4	2
5-HETE	24	84	9	15	10
5-HEPE	37	87	10	16	10
7-HDHA	43	90	15	44	19
5*S*,6*R*-diHETE	9	88	3	3	3
15-HETE	221	133	211	204	214
15-HEPE	186	126	184	209	177
17-HDHA	205	119	129	175	122
12-HETE	164	113	103	136	114
12-HEPE	137	109	102	133	110
14-HDHA	133	99	75	108	83

Figure S4. Human monocyte-derived macrophages were pre-treated with test compounds (10 µM) or vehicle 15 min prior stimulation. Cells were then stimulated with S. aureus for 90 min. Data are expressed as percentage of the mean of the 100% control DMSO of n=3 experiments.

Materials and methods

Computational procedures

The structures of the reagents, namely all commercially available acyl chlorides and boronic acids at Sigma-Aldrich, Merck, were converted from 2D structures to 3D structures suitable for the docking stage of the process, using LigPrep software.[1] Then, the compounds were prepared using Reagent Preparation: in the acyl chlorides, a cleavage between the chloride and the carbonyl group was applied in order to select the carbonyl moiety as a building block, while the acidic moiety was removed in the boronic acids. The final .bld files were combined with the scaffolds obtaining the novel library. LigPrep performed calculation increased the number to 424,202 molecules for the library, and final energy minimization of the library with Optimized Potentials for Liquid Simulations (OPLS) 2005 force field was performed. After the application of QikProp[2] and LigFilter, the final library contained 15,412 compounds.

The successive application of LigPrep, QikProp and LigFilter[2] software was used to assess the final pool of molecules for the *in silico* binding prediction towards mPGES-1. Ligprep software was used to prepare the starting combinatorial library, taking into account all possible tautomers and protonation states at pH = 7.4 ± 1. Then, the pharmacokinetic properties, physically significant descriptors, and pharmaceutically parameters for prediction of absorption, distribution, metabolism, and excretion (ADME) were calculated by QikProp software. After that, thanks to the LigFilter module (see Table S1), only the compounds characterized by "drug-like" properties were considered for the next molecular docking calculation, obtaining a final library of ~ 4.2×10^5 2-amino-thiadiazole-based entities using a virtual screening workflow (VSW, Glide software).[3]

VSW consisted of three rounds of experiments: 1) High-Throughput Virtual Screening (HTVS) precision mode of Glide for a first enrichment from the starting library of compounds with a high fastness; 2) Standard Precision (SP) for the analysis of the 60% top-ranked poses of HTVS filtered according to docking score values, which overcomes the first step in both sampling and scoring

performances; 3) Extra-Precision (XP) for the analysis of the 70% top-ranked poses of SP using the Glide mode experiment, the final and most accurate docking step. Finally, specific filters on selected docking poses were applied using the pose filter tool of Maestro, setting the key interactions as a qualitative filter. Furthermore, the final selection of the most promising molecules was also optimizing considering computational tools: a) SwissADME,[4] for filtering the compound displaying the features of "Pan-Assay Interference Compounds"; b) detailed analysis of the other specific pharmaceutically relevant properties obtained by QikProp[2] software (Table S1).

Trimer structure of murine mPGES-1. In order to be consistent with the biological assays performed, the 3D protein model of murine mPGES-1 was built, through a homology modelling procedure, due to its absence of any solved structure in the Protein Data Bank. Sequence alignment was performed on the amino acid sequence of human mPGES-1 (accession number of O14684) and murine mPGES-1 (accession number of Q9JM51) by using the Basic Local Alignment Search Tool (BLAST) of Uniprot server (http://www.uniprot.org/align/).[5] The aligned sequence indicates that human mPGES-1 is highly homologous to murine mPGES-1, with the sequence identity being 79% and the sequence similarity being 84%

The crystal structure of human mPGES-1 available in the Protein Data Bank (PDB code: 4BPM; https://www.rcsb.org/structure/4BPM) was used as a template. The protein model was generated using Prime software;[6-8] afterwards, the quality of the generated model was assessed through PROCHECK (Figure S5)[9].

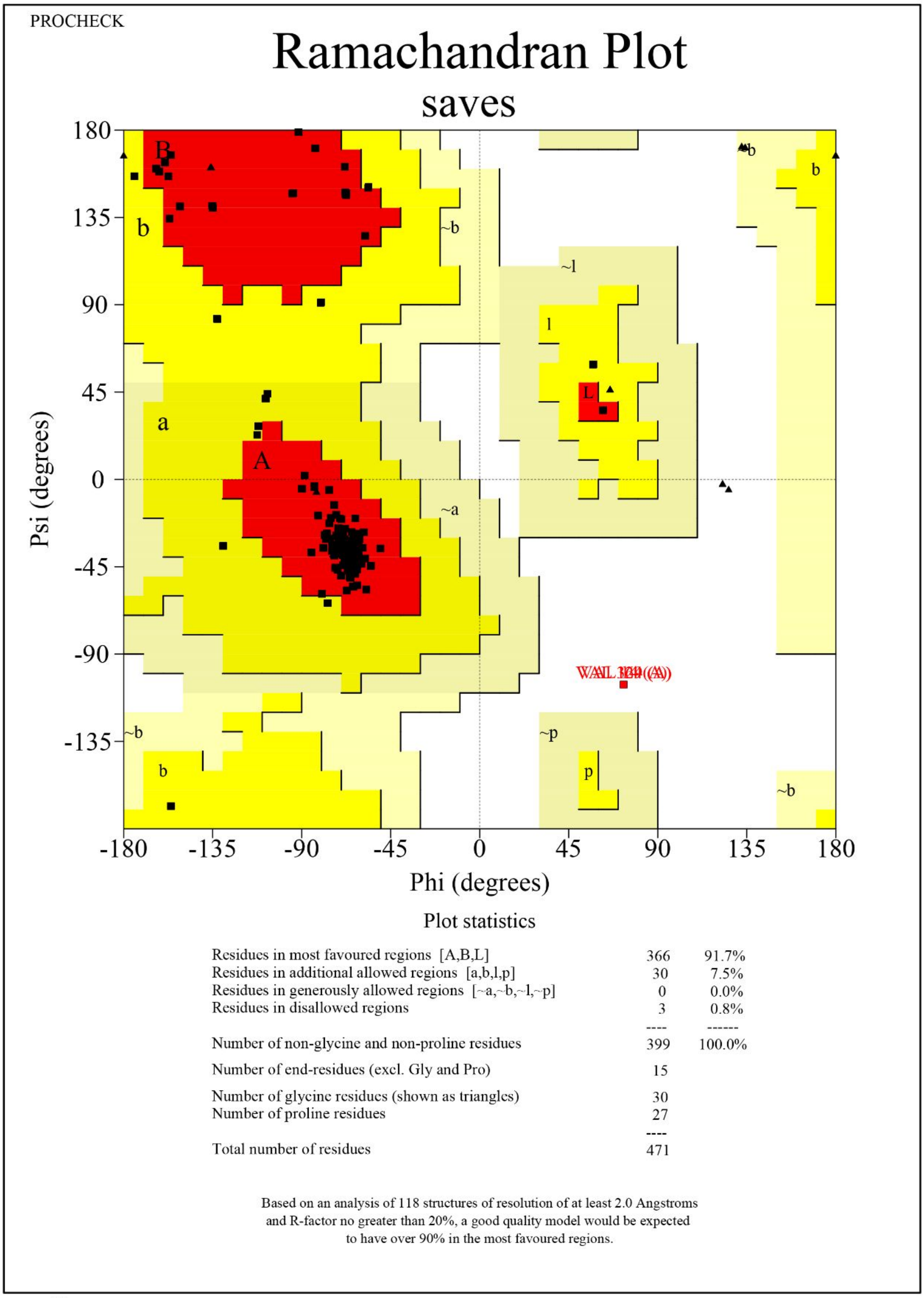

Figure S5. The Ramachandran plot showing the phi-psi torsion angles for all residues (except those at the chain termini) for the protein model murine mPGES-1 built by homology modeling. Glycine residues are separately identified by triangles as these are not restricted to the regions of the plot appropriate to the other sidechain types. The colouring/shading on the plot represents the different regions described in Morris et al. (1992)[9]: the darkest areas (here shown in red) correspond to the "core" regions representing the most favourable combinations of phi-psi values. Ideally, one would hope to have over 90% of the residues in these "core" regions; in this protein model, this value was reached, thus highlighting the reliability of the built structure.

Molecular docking experiments of the most active compounds (**3**, **6**, **7** and **9**, Figure S6) towards the *in silico* model of murine mPGES-1 were performed using Glide software (Schrödinger Suite),[10-13] using the Standard Precision [SP] mode. In detail, 10,000 poses were kept in the starting phase of docking for energy minimization, setting the scoring window for keeping the initial poses to 400.0 and a scaling factor of 0.8 related to van der Waals radii, with a partial charge cutoff of 0.15, basing on a 0.5 kcal/mol rejection cutoff for the obtained minimized poses. Eventually, 20 maximum number of poses for each compound were saved in the output file. From the analysis of the most representative binding poses of our hits (**3**, **6**, **7** and **9**, Figure S6) in the above-mentioned 3D model of the murine mPGES-1, all of them are able to fit in the pharmacological site of interest, where the interaction with the key residues could be compatible with an inhibitor of this protein.

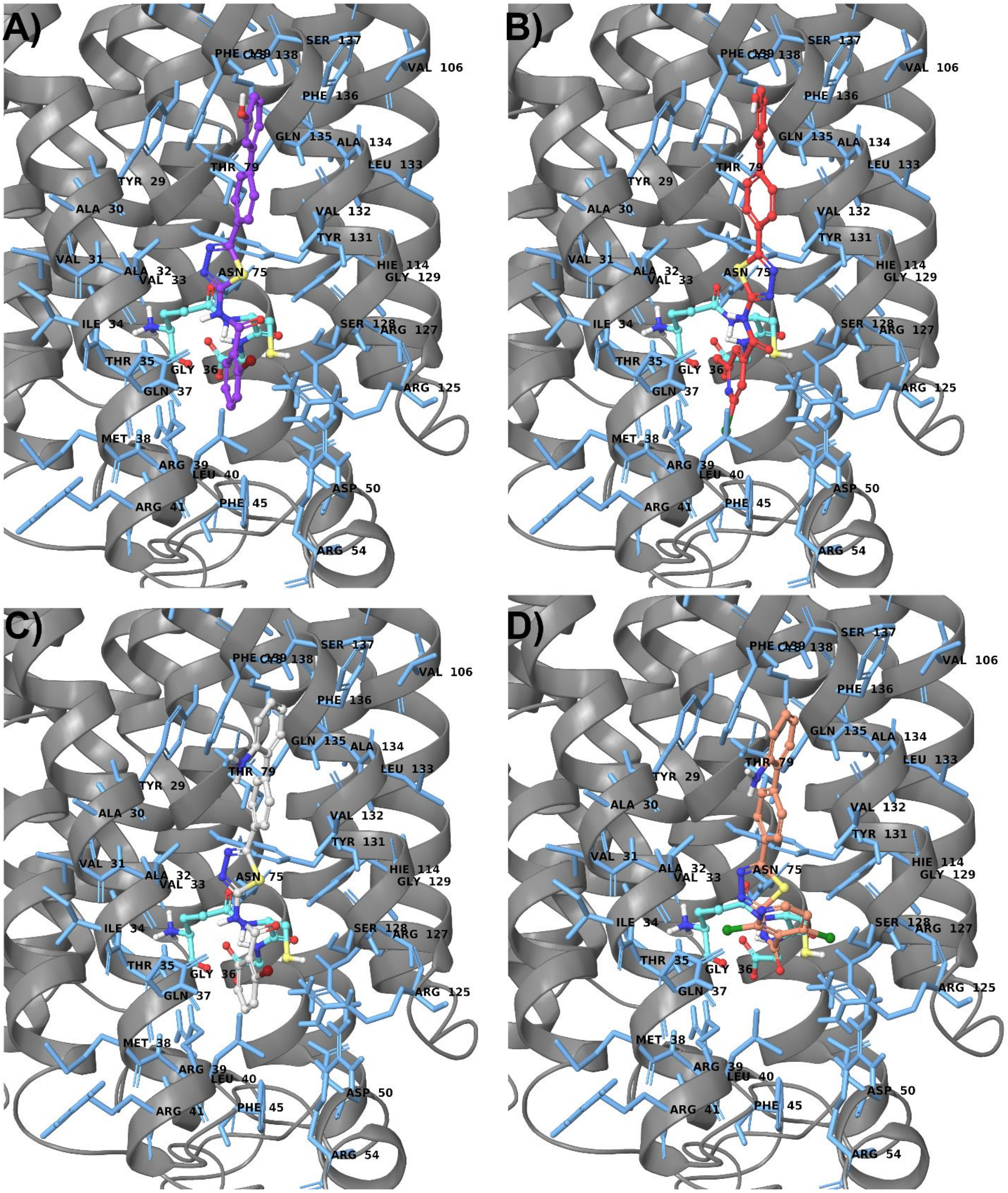

Figure S6. A) **3** (colored by atom type: C violet, O red, N blue, polar H light grey), B) **6** (colored by atom type: C red, O red, N blue, polar H light grey); C) **7** (colored by atom type: C white, O red, N blue, polar H light grey); D) **9** (colored by atom type: C salmon pink, O red, N blue, polar H light grey) in complex with the model of murine mPGES-1 (secondary structure and key residues are reported as grey ribbon and blue sticks.

General synthetic procedures

All chemicals were purchased from commercial suppliers and used as received unless otherwise indicated. No unexpected or unusually high safety hazards were encountered during experimental procedures. ^{1}H NMR spectra were recorded on 400 MHz Bruker Avance instrument. Chemical shifts are expressed in δ ppm and are calibrated to the residual solvent peakas internal reference. Coupling constants (*J*) are reported in Herz. Multiplicities are reported as follows: s, singlet; d, doublet; t, triplet; m, multiplet; dd, doublet of doublets. ^{13}C NMR spectra were obtained at 100 MHz and referenced to the internal solvent signal. DEPTQ experiments (dept polarization transfer with decoupling during acquisition using shaped pulse for 180 degree pulse on f1channel) were acquired at 100 MHz. ESI-MS spectra were carried out in positive mode on a high-resolution mass spectrometer (HRMS) Q Exactive™ Plus Hybrid Quadrupole-Orbitrap™ Mass Spectrometer (Termo Fisher Scientific). Reactions were monitored on silica gel 60 F254 plates (Merck), and the spots were visualized under UV light. Analytical and semi-preparative reversed-phase HPLC was performed on Agilent Technologies 1200 Series high performance liquid chromatography using a Synergi Fusion C18 reversed-phase column (250 x 4.60mm, 4μ, 80 Å, flow rate = 1 mL/min; 250 x 10.00mm, 10μ, 80 Å, flow rate = 4 mL/min respectively, Phenomenex®). The binary solvent system (A/B) was as follows: 0.1% TFA in water (A) and 0.1% TFA in CH_3CN (B); the absorbance was detected at 280 nm. All final compounds were purified to ≥95% purity by HPLC analysis and were fully characterized by HRMS, and NMR spectra.

Synthesis of 4'-(5-amino-1,3,4-thiadiazol-2-yl)-(1,1'-biphenyl)-3-ol (10)

2-Amino-5-(4-bromophenyl)-1,3,4-thiadiazole (1.15 mmol.), 3-hydroxyphenylboronic acid (1.72 mmol) and (1,1′-bis(diphenylphosphino)ferrocene)dichloropalladium(II) (20 mol %), were added to a solution of 1,4-dioxane (70%) and water (30%) (25 mL) previously degassed and the mixture was stirred at r.t. for 30 min. Then a solution of aqueous K_2CO_3 1M (3.45 mmol) was added, and the reaction was stirred at 80 °C overnight under argon. The mixture was extracted with ethyl acetate,

and the organic layer was washed with brine, and dried over Na_2SO_4. The solvent was evaporated under reduced pressure, and the residue was purified by flash chromatography on silica gel, eluting with 20% AcOEt in petroleum ether, to afford **10** (yield 63%).^{1}H NMR ($CDCl_3$, 400 MHz): 9.35 (s, 1H, OH); 7.57 (d, *J* = 8.4 Hz, 2H); 7.44 (d, *J* = 8.4 Hz, 2H); 7.03 (t, *J* = 7.9 Hz, 1H); 6.86 (d, *J* = 7.7 Hz, 1H); 6.81 (m, 1H); 6.54 (dd, *J* = 8.0, 1.6 Hz, 1H)

General procedure for the synthesis of 1, 2, 3, 4

A solution of **10** (0.15 mmol) and pyridine (4.5 mmol) in dry acetonitrile (5 mL) at 0°C was treated with chlorotrimethylsilane (1.5 mmol). After addition, reaction was stirred for 2h at room temperature, then the appropriate acyl chloride was added (0.22 mmol). The mixture was stirred for 2 h, then was quenched with HCl 0.5 M (5 mL) and extracted with AcOEt (3x 5 mL). Organic phase was washed with brine, dried (Na_2SO_4), and the solvent was removed under reduced pressure. The residue was then purified by flash chromatography on silica gel or reversed-phase HPLC.

N-(5-(3'-Hydroxy-(1,1'-biphenyl)-4-yl)-1,3,4-thiadiazol-2-yl)-3-methylfuran-2-carboxamide (1)

1 was prepared using 3-methylfuran-2-carbonyl chloride as acyl chloride. The product was purified by semi-preparative reversed-phase HPLC (gradient conditions: from 5% B to 50% B in 5 min, increased to 100% B in 40 min; t_R= 19.6 min) to afford **1** (92 % yield)

^{1}H NMR (DMSO-d_6, 400 MHz): 8.04 (d, *J* = 8.1 Hz, 2H); 7.90 (d, *J* = 1.7 Hz, 1H); 7.79 (d, *J* = 8.1 Hz, 2H); 7.31 (t, *J* = 7.9 Hz, 1H); 7.17 (d, *J* = 7.7 Hz, 1H); 7.11 (s, 1H); 6.82 (dd, *J* = 8.1, 2.2 Hz, 1H); 6.68 (d, *J* = 1.7 Hz, 1H); 2.40 (s, 3H).

^{13}C NMR (DMSO-d_6, 100 MHz): 158.4; 158.1; 158.0; 157.8; 157.5; 145.7; 142.3; 140.5; 131.4; 130.2; 129.2; 127.5 (4C); 117.6; 116.1; 115.2; 113.5; 11.2.

HR-MS: *m/z* calcd for $C_{20}H_{15}N_3O_3SNa$ $(M+Na)^+$ 400.0732; found 400.0734.

2-Chloro-N-(5-(3'-hydroxy-(1,1'-biphenyl)-4-yl)-1,3,4-thiadiazol-2-yl)-6-methylisonicotinamide (2)

2 was prepared using 2-chloro-6-methylpyridine-4-carbonyl chloride as acyl chloride. The product was purified by flash chromatography on silica gel, eluting with 40% AcOEt in petroleum ether, to afford **2** (yield 81%).

^{1}H NMR (DMSO-d_6, 400 MHz): 9.67 (s, 1H, OH); 8.05 (d, *J* = 8.0 Hz, 2H); 7.93 (s, 1H); 7.89 (s, 1H); 7.78 (d, *J* = 8.0 Hz, 2H); 7.31 (t, *J* = 7.9 Hz, 1H); 7.17 (d, *J* = 7.9 Hz, 1H); 7.11 (s, 1H); 6.83 (d, *J* = 7.9 Hz, 1H); 2.57 (s, 3H).

^{13}C NMR (DMSO-d_6, 100 MHz): 161.5; 160.3; 158.0; 157.8; 150.0; 143.89; 143.83; 142.3; 140.5; 130.2; 129.3; 127.5 (4 C); 121.1; 119.8; 117.6; 115.2; 113.5; 25.8.

HR-MS: *m/z* calcd for $C_{21}H_{16}ClN_4O_2S$ $(M+H)^+$ 423.0683; found 423.0685.

2-Bromo-N-(5-(3'-hydroxy-(1,1'-biphenyl)-4-yl)-1,3,4-thiadiazol-2-yl) benzamide (3)

3 was prepared using 2-bromobenzoyl chloride as acyl chloride. HPLC gradient conditions: from 5% B to 40% B in 5 min, increased to 60% B in 30 min; t_R= 9.3 min. Yield 58%.

^{1}H NMR (DMSO-d_6, 400 MHz): 9.67 (s, OH, 1H); 8.05 (d, *J* = 8.0 Hz, 2H); 7.77 (m, 3H); 7.68 (d, *J* = 7.4 Hz, 1H); 7.50 (m, 2H); 7.31 (t, *J* = 7.9 Hz, 1H); 7.17 (d, *J* = 7.7 Hz, 1H); 7.11 (s, 1H); 6.82 (d, *J* = 8.0 Hz, 1H).

^{13}C NMR (DMSO-d_6, 100 MHz): 166.2; 162.0; 157.9; 157.7; 142.4; 140.5; 138.3; 133.0; 132.3; 130.2; 130.0; 129.2; 127.8; 127.6 (2 C); 127.5 (2 C); 119.3; 117.6; 115.2; 113.5.

HR-MS: *m/z* calcd for $C_{21}H_{14}BrN_3O_2SNa$ $(M+Na)^+$ 473.9888; found 473.9891.

2,5-Difluoro-N-(5-(3'-hydroxy-(1,1'-biphenyl)-4-yl)-1,3,4-thiadiazol-2-yl) benzamide (4)

4 was prepared using 2,5-difluorobenzoyl chloride as acyl chloride. HPLC gradient conditions: from 5% B to 65% B in 5 min, increased to 100% B in 45 min; t_R= 14.1 min. Yield 75%.

^{1}H NMR (DMSO-d_6, 400 MHz): 7.92 (d, *J* = 8.3 Hz, 2H); 7.69 (m, 2H); 7.61 (m, 1H); 7.27 (t, *J* = 7.9 Hz, 1H); 7.21 (m, 2H); 7.13 (d, *J* = 7.6 Hz, 1H); 7.08 (s, 1H); 6.78 (dd, *J* = 8.2, 2.4 Hz, 1H).

^{13}C NMR (DMSO-d_6. 100 MHz): 162.1; 160.2 (d, J = 210 Hz); 160.0; 158.6; 155.9 (d, J = 246 Hz); 151.0; 141.2; 140.8; 130.5; 129.7; 127.8 (4 C); 124.8; 121.6; 120.2; 119.5 (dd, J = 25.0, 8.4 Hz); 118.9 (m); 118.5 (dd, J = 24.3, 8.4 Hz); 118.4 (d, J = 25.9 Hz).

HR-MS: *m/z* calcd for $C_{21}H_{14}F_2N_3O_2S$ $(M+H)^+$ 410.0775; found 410.0778.

Synthesis of tert-butyl (4'-(5-amino-1,3,4-thiadiazol-2-yl)-(1,1'-biphenyl)-2-yl) carbamate (11)

2-amino-5-(4-bromophenyl)-1,3,4-thiadiazole (0.35 mmol.), 2-(N-Boc-amino)phenylboronic acid pinacol ester (0.52 mmol) **II** and (1,1′-bis(diphenylphosphino)ferrocene)dichloropalladium(II) (20 mol %), were added to 30 mL of a degassed mixture of 1,4-dioxane (70%) and water (30%). After 30 min a degassed aqueous solution of K_2CO_3 1M (1.0 mmol) was added and the reaction mixture was stirred at 80 °C for 16 h under argon. The reaction mixture was extracted with ethyl acetate, then the organic layer was washed with brine, and dried over Na_2SO_4. The solvent was evaporated under reduced pressure, and the residue was purified by flash chromatography on silica gel, eluting with 50% AcOEt in petroleum ether, to afford **11** (yield 72%).

^{1}H NMR (CD_3OD 400 MHz): 7.83 (d, J = 7.9 Hz, 2H); 7.53 (m, 3H); 7.35 (m, 2H); 7.27 (m, 1H); 1.36 (s, 9H).

General procedure for the synthesis of 5, 6, 7, 8, 9

A solution of **11** (0.1 mmol) and pyridine (0.15 mmol) in dry acetonitrile (5 mL) was treated with the proper acyl chloride (0.15 mmol) at room temperature. The mixture was stirred for 2 h, then was quenched with HCl 0.5 M (5 mL) and extracted with AcOEt (3x 5 mL). Organic phase was washed with brine, dried (Na_2SO_4) and the solvent removed under reduced pressure. The crude product was then dissolved in 5 ml of TFA/ CH_3CN 1:1 and stirred at room temperature. After 2 h the solvent was removed under reduced pressure and the residue was purified by reversed-phase HPLC.

N-(5-(2'-Amino-(1,1'-biphenyl)-4-yl)-1,3,4-thiadiazol-2-yl)-3-methylfuran-2-carboxamide (5)

5 was prepared using 3-methylfuran-2-carbonyl chloride as acyl chloride. HPLC gradient conditions: from 5% B to 100% B in 50 min; t_R = 27.0 min. Yield 26% over 2 steps.

^{1}H NMR (CD_3OD, 400 MHz): 8.02 (d, *J* = 8.3 Hz, 1H); 7.61 (d, *J* = 1.7 Hz, 0H); 7.53 (d, *J* = 8.3 Hz, 1H); 7.32 (m, 2H); 7.20 (m, 1H); 6.50 (d, *J* = 1.7 Hz, 1H); 2.38 (s, 3H).

^{13}C NMR (DMSO-d_6, 100 MHz): 161.2; 158.4; 156.9; 145.7; 142.8; 141.6; 140.3; 131.4; 130.2; 129.7 (2C); 128.9; 128.7; 127.4 (2C); 126.3; 118.7; 116.9; 116.1; 11.2.

HR-MS: *m/z* calcd for $C_{20}H_{17}N_4O_2S$ $(M+H)^+$ 377.1072; found 377.1074.

N-(5-(2'-Amino-(1,1'-biphenyl)-4-yl)-1,3,4-thiadiazol-2-yl)-2-chloro-6-methylisonicotinamide (6)

6 was prepared using 2-chloro-6-methylpyridine-4-carbonyl chloride as acyl chloride. HPLC gradient conditions: from 5% B to 100% B in 45 min; t_R = 29.1 min). Yield 31% over 2 steps.

^{1}H NMR (DMSO-d_6, 400 MHz): 8.06 (d, *J* = 8.0 Hz, 2H); 7.94 (s, 1H); 7.90 (s, 1H); 7.62 (d, *J* = 8.0 Hz, 2H); 7.09 (m, 2H); 6.83 (d, *J* = 8.0 Hz, 1H); 6.72 (t, *J* = 7.4 Hz, 1H); 2.58 (s, 3H).

^{13}C NMR (DMSO-d_6, 100 MHz): 162.1; 160.5; 158.1; 157.8; 150.1; 144.5; 143.0; 142.3; 130.1; 129.7 (2 C); 128.8; 128.3; 127.5 (2 C); 125.1; 121.0; 119.8; 117.5; 116.0; 23.8.

HR-MS: *m/z* calcd for $C_{21}H_{17}ClN_5OS$ $(M+ H)^+$ 422.0842; found 422.0847.

N-(5-(2'-Amino-(1,1'-biphenyl)-4-yl)-1,3,4-thiadiazol-2-yl)-2-bromobenzamide (7)

7 was prepared using 2-bromobenzoyl chloride as acyl chloride. HPLC gradient conditions: from 5% B to 100% B in 45 min; t_R = 26.4 min. Yield 36% over 2 steps.

^{1}H NMR (CD_3OD, 400 MHz): 8.14 (d, *J* = 8.4 Hz, 2H); 7.76 (d, *J* = 7.9 Hz, 1H); 7.63 (d, *J* = 8.0 Hz, 3H); 7.51 (m, 5H); 7.43 (d, *J* = 7.6 Hz, 1H).

^{13}C NMR (CD_3OD, 100 MHz): 168.2; 164.9; 160.6; 141.2; 137.6; 135.7; 134.8; 133.7; 132.8; 131.70; 131.3; 130.9; 130.5; 129.2; 129.1; 128.7; 123.7; 120.9.

HR-MS: *m/z* calcd for $C_{21}H_{16}BrN_4OS$ (M+H)$^+$ 451.0228; found 451.0230; *m/z* calcd for $C_{21}H_{15}BrN_4OSNa$ (M+Na)$^+$ 473.0048; found 473.0050.

N-(5-(2'-Amino-(1,1'-biphenyl)-4-yl)-1,3,4-thiadiazol-2-yl)-2,5-difluorobenzamide (8)

8 was prepared using 2,5-difluorobenzoyl chloride as acyl chloride. HPLC gradient conditions: from 5% B to 100% B in 50 min; t_R= 29.6 min. Yield 53% over 2 steps.

^{1}H NMR (CD_3OD, 400 MHz): 8.12 (d, *J* = 8.3 Hz, 2H); 7.62 (m, 3H); 7.39 (m, 4H); 7.26 (m, 2H).

^{13}C NMR (DMSO-d_6 100MHz): 162.1; 160.1 (d, *J* = 210 Hz); 158.9; 158.6; 155.8 (d, *J* = 245 Hz); 147.9; 141.2; 134.4; 129.7; 128.4; 128.3; 127.7 (4 C);120.5; 119.4 (dd, *J* = 25.0, 8.2 Hz); 118.8 (m); 118.5 (dd, *J* = 24.0, 8.3 Hz); 118.4 (d, *J* = 25.7 Hz); 116.8.

HR-MS: *m/z* calcd for $C_{21}H_{15}F_2N_4OS$ (M+H)$^+$ 409.0935; found 409.0933.

N-(5-(2'-Amino-(1,1'-biphenyl)-4-yl)-1,3,4-thiadiazol-2-yl)-2,4-dichloronicotinamide (9)

9 was prepared using 2,4-dichloropyridine-3-carbonyl chloride as acyl chloride. HPLC gradient conditions: from 5% B to 100% B in 45 min; t_R = 32.0 min). Yield 42% over 2 steps.

^{1}H NMR (CD_3OD, 400 MHz): 8.50 (d, *J* = 5.5 Hz, 1H); 8.14 (d, *J* = 7.8 Hz, 2H); 7.67 (d, *J* = 5.5 Hz, 1H); 7.63 (d, *J* = 7.8 Hz, 2H); 7.46 (m, 2 H); 7.37 (m, 2H).

^{13}C NMR (CD_3OD, 100 MHz): 165.7; 163.6; 160.3; 153.0; 150.1; 145.2; 142.2; 134.9; 134.9; 132.9; 131.2; 131.7; 131.2; 129.6; 127.8; 125.9; 123.2.

HR-MS: *m/z* calcd for $C_{20}H_{14}Cl_2N_5OS$ (M+ H)$^+$ 442.0296; found 442.0304.

Cell-free mPGES-1 activity assay

Microsomes were obtained from A549 cells previously stimulated with IL-1β (1 ng/ml) for 48 h, and were used as source for mPGES-1. After sonication, differential centrifugations were applied to the resulting homogenate, specifically at 10,000 × g for 10 min and 174,000 × g for 1 h at 4 °C. The pellet was resuspended by adding 1 ml homogenization buffer (0.1 M potassium phosphate buffer, pH 7.4, 1 mM phenylmethanesulfonyl fluoride, 60 μg/mL soybean trypsin inhibitor, 1 μg/mL leupeptin, 2.5

mM glutathione, and 250 mM sucrose) and the protein concentration was defined. Potassium phosphate buffer (0.1 M, pH = 7.4) plus glutathione (2.5 mM) was added to dilute microsomes, which were then seeded in a 96-well plate. Test compounds **1-9 (**10 µM) or DMSO (1%) were added and preincubated for 15 min on ice. MK886 (10 µM) and MD-52 (1 µM) were used as reference drugs. The reactions started by adding 20 µM of PGH_2, and 1 min later, 100 µl of a solution containing $FeCl_3$ (40 mM), citric acid (80 mM), and 11β-PGE_2 (10 µM) were added for stopping the conversion of PGH_2. After the extraction of PGE_2 and 11β-PGE_2 (internal stardard) by solid-phase extraction, the quantity of product formation (PGE_2) was determined by RP-HPLC.[114]

Cell-free 5-LO activity assay

Human recombinant 5-LO was expressed in *E.coli* BL21 transformed with pT3-5-LO plasmid at 30 °C overnight[15].[2] A lysis buffer (50 mM triethanolamine, pH = 8.0, 5 mM EDTA, 1 mM phenylmethanesulfonyl fluoride, 60 µg/mL soybean trypsin inhibitor, 2 mM dithiothreitol and 1 mg/mL lysozyme) was added. Cells were homogenized by sonication (3 × 15 s), a centrifugation of 40,000 × g for 20 min at 4 °C was applied, and the supernatant was collected. An ATP-agarose column was used to obtain the pure enzyme, which was diluted with PBS buffer (containing 1 mM EDTA). Afterwards, an amount of 0.5 µg of 5-LO in 1 mL PBS with 1 mM EDTA was pre-incubated with the test compounds (**3, 6, 7, 9**) or vehicle (0.1% DMSO) on ice over 10 min, and then stimuli were added (20 µM AA and 2 mM $CaCl_2$). After 10 min at 37 °C, 1 mL of ice-cold methanol, and then 530 µl PBS plus HCl and PGB_1 (internal standard) were added. Finally, a solid-phase extraction was performed using C18 RP-columns (100 mg, UCT, Bristol, PA, USA) and RP-HPLC was used to quantify 5-LOX products.[3] [16] The 5-LO inhibitor zileuton (3 µM) was used as positive control.

Cell-free COXs activity assay

Isolated COX-1 (ovine) and COX-2 (human) were used for the evaluation of the activity of test compounds **3**, **6**, **7**, and **9** on cyclooxygenases. COXs were diluted using Tris buffer (100 mM, pH =

8) plus 5 mM glutathione, 100 μM EDTA and 5 μM hemoglobin) to a final concentration of 50 U/mL for COX-1 and 20 U/mL for COX-2, and pre-incubated with test compounds **3**, **6**, **7**, and **9** (10 μM) or vehicle (0.1% DMSO) over 5 min at rt. After 1 min at 37 °C, reactions were started after addition of arachidonic acid to a final concentration of 5 μM for COX-1 and 2 μM for COX-2. After 5 min at 37 °C, 1 mL of ice-cold methanol was added for stopping the reactions on ice. Then, PGB_1 as internal standard and 530 μl PBS plus HCl were added, and solid-phase extraction was performed as reported before.[4-6, 17-19] COXs product formation (12-HHT) was determined using RP-HPLC.[4-6, 17-19] Indomethacin (10 μM) and celecoxib (5 μM) were used as reference drugs.

Expression, purification and activity assay of human recombinant sEH

Human recombinant sEH was expressed in Sf9 cells and was infected using a baculovirus, provided by Dr. B. Hammock, University of California, Davis, CA. After 72 hours, cells were pelleted and sonicated (3 × 10 sec) at 4 °C in lysis buffer (50 mM $NaHPO_4$, pH = 8, 300 mM NaCl, 10% glycerol, 1 mM EDTA, 1 mM phenylmethanesulfonyl fluoride, 10 μg/mL of leupeptin, and 60 μg/mL of soybean trypsin inhibitor). Lysates were centrifuged over 60 min at 4 °C (100,000 × g), and supernatants were collected and submitted to a benzylthio-sepharose-affinity chromatography for sEH purification by elution with 4-fluorochalcone oxide in PBS plus 1 mM DTT and 1 mM EDTA.[720] Bio-Rad protein detection kit (Bio-Rad Laboratories, Munich, Germany) was used for total protein on dialyzed and concentrated (Millipore Amicon-Ultra-15 centrifugal filter) enzyme solution and a fluorescence-based assay was used to define the epoxide hydrolase activity.[5,618, 19] Then, sEH was diluted in Tris buffer (25 mM, pH = 7) supplemented with 0.1 mg/mL of BSA to an appropriate enzyme concentration and pre-incubated with test compounds **3**, **6**, **7**, **9** (10 μM) or vehicle (0.1% DMSO) for 15 min at rt. 50 μM 3-phenyl-cyano(6-methoxy-2-naphthalenyl)methyl ester-2-oxiraneacetic acid (PHOME), a non-fluorescent compound, was added for starting the reaction. PHOME is enzymatically converted into a fluorescent compound, 6-methoxy-naphtaldehyde, detectable by using a spectrofluorometer setting λ_{em} = 465 nm, λ_{ex} = 330 nm. After 60 min, $ZnSO_4$

(200 mM) was added for stopping the reaction, and then fluorescence was detected. AUDA (12-(3-adamantan-1-yl-ureido)-dodecanoic acid) (100 nM) was used as reference drug.

Cells

Human neutrophils and monocytes were freshly isolated from leukocyte concentrates obtained from the Institute of Transfusion Medicine, University Hospital Jena. Donors were healthy adult volunteers and gave written consent after they were informed about the use of their blood samples for the study, after the approval of the ethical commission (EC). Importantly, all procedures were performed in line with the relevant guidelines and regulations.

Neutrophils were isolated[821] by dextran sedimentation, centrifugation on lymphocyte separation medium (LSM 1077, PAA, Coelbe, Germany), and hypotonic lysis of erythrocytes, and were resuspended in PBS containing 0.1% of glucose to a final cell density of 5×10^6 cells/mL.

Concerning monocytes, they were separated from peripheral blood mononuclear cells (PBMC) by adherence to cell culture flasks (Greiner Bio-one, Nuertingen, Germany) for 1.5 h, at 37 °C and 5% CO_2 in RPMI 1640 containing L-glutamine (1 mM), heat-inactivated FCS (10%), penicillin (100 U/mL) and streptomycin (100 µg/mL), followed by cell-scraping and resuspension in PBS.

In order to differentiate monocytes into M1 macrophages, freshly isolated monocytes were kept at 37 °C in RPMI medium (supplemented with 10% FCS, 2 mM *L*-glutamine, 100 U/mL penicillin and 100 µg/mL streptomycin) containing 20 ng/mL GM-CSF (Peprotech, Hamburg, Germany) for 6 days. Polarization towards a pro-inflammatory M1 phenotype was obtained by stimulation of GM-CSF-treated macrophages ($M0_{GM\text{-}CSF}$) with 100 ng/mL lipopolysaccharide (LPS) and 20 ng/mL interferon (IFN)-γ (Peprotech, Hamburg, Germany) for 48 h.

Cell lines

A549 cells were purchased from Cell Application Inc., Sigma-Aldrich, Merck (Darmstadt, Germany) maintained in DMEM supplemented with 10% heat-inactivated fetal bovine serum (Invitrogen,

Carslbad, CA, USA), in 5% CO_2 humid atmosphere. The cells were subcultured every 2 days and were tested for Mycoplasma using PCR analysis.Inhibition of PGE_2 production in cells

A549 cells were plated at 10.000 cells/well in DMEM with 10% FBS-medium in a 96-well plate; after overnight incubation, the cells were treated with 5 and 10 μM of **3, 6**, **7**, **9** compounds in DMEM 1%FBS and IL-1β (10 ng/ml) to upregulate PG synthase expression.

After 24 h, the amount of PGE_2 released in the supernatant was evaluated by a commercially available enzyme immunoassay kit (Prostaglandin E2 EIA kit Monoclonal, Cayman Chemical), and at the same time was evaluated cell viability by MTT using (3-4,5-dimethyldiazol-2-yl)-2,5-diphenyl tetrazolium bromide reagent (Sigma-Aldrich). Following the treatment, 20 μL of MTT (5 mg/mL in PBS) was added, and the cells were incubated for additional 3 h at 37°C. The formazan crystals thus formed were dissolved in 100 μl of buffer containing 50% (v/v) N,N-dimethylformamide, 20% SDS (pH 4.5). The absorbance was measured at 570 nm with a Multiskan™ GO Microplate Spectrophotometer (Thermo Fisher Scientific, USA).

Cell viability assay on A549 cell line

MTT conversion assay was performed on A549 cell line after incubation with test compounds for evaluating cell viability. The cells (2 x 10^4) were seeded in triplicate in 96 well/plates and incubated with compounds **3, 6**, **7**, **9** (10 μM) or vehicle DMSO 0.1% (v/v) for 48 h in DMEM (37 °C, 5% CO_2). MTT (5 mg/mL) was added, and after 1 h (37 °C, 5% CO_2) the medium was replaced with DMSO (100 μL per well). Finally, formazan formation was detected by the measurement of absorbance at 570 nm.

Cell viability assay on monocytes

Acute cytotoxicity of compounds **3, 6, 7, 9** was analyzed on human monocytes. Cells (0.2×10^6 per well) were seeded in 100 μL buffer on 96-well plates and treated with the test compounds (at a concentration of 10 μM), triton (0.1%, as positive control) or vehicle (0.5% of DMSO) over 24 hours

(37 °C, 5% CO_2). MTT (5 mg/mL) was added and after 2 h (37 °C, 5% CO_2) cells were lysed by SDS treatment (10%, pH = 4.5). After 17 hours, formazan formation was detected by measurement of absorbance at 570 nm.

Determination of 5-LO products in neutrophils

Freshly isolated neutrophils were resuspended in PBS buffer containing 0.1% glucose and 1 mM $CaCl_2$ to a final cell density of 5 × 10^6. Cells were pre-incubated with compounds **3, 6, 7, 9** or DMSO vehicle (0.1%) at 37 °C for 10 min. Then, 2.5 µM Ca^{2+}-ionophore A23187 (with or without supplementation of 20 µM of arachidonic acid) was added as stimulus, and cells were left at 37 °C for 10 min. 1 mL of ice-cold methanol was used to stop the reaction of 5-LO product formation on ice, and 530 µL of PBS plus HCl and PGB_1 (as internal standard) were added. Finally, cells were centrifuged (2000 x g) over 10 min at room temperature, and 5-LO products were extracted by solid-phase extraction, and they were quantified by RP-HPLC as described above. Zileuton (3 µM) was used as reference drug.

Determination of LO products in human macrophages

Human M1 macrophages (2 x 10^6 /mL) were pre-treated with test compounds or vehicle (DMSO, 0.1%) for 15 min. Afterward, 1% *Staphylococcus aureus* 6850wt-conditioned medium (SACM) was added for 90 minutes at 37 °C in order to induce LO product formation. The reaction was stopped by transferring supernatants (1 mL) into 2 mL ice-cold MeOH. After addition of the deuterated LM standards (200 nM d8-5S-HETE, d4-LTB_4, d5-LXA_4, d5-RvD2, d4-PGE_2 and 10 µM d8-AA; Cayman Chemical/Biomol GmbH, Hamburg, Germany), samples were kept at −20 °C for 60 min and then centrifuged (1,200×g, 4°C, 10 min). Solid-phase C18 cartridges were equilibrated with 6 mL methanol before the addition of 6 mL H_2O. Then, 9 mL acidified H_2O (pH 3.5, HCl) was added, and the samples were loaded onto the conditioned C18 columns that were subsequently washed with 6 mL H_2O, followed by 6 mL *n*-hexane. The LM were eluted with 6 mL of methyl formate, samples were brought to dryness using an evaporation system (TurboVap LV, Biotage) and immediately

resuspended in methanol-water (50:50 vol/vol) for UPLC-MS-MS automated injections. The UPLC-MS-MS system consisted of an Acquity UPLC BEH C18 column (1.7 µm, 2.1 × 50 mm, Waters) and an Acquity™ UPLC (Waters) as well as a QTRAP 5500 mass spectrometer (Sciex, Darmstadt, Germany) equipped with an electrospray ionization source. The QTrap 5500 was operated in negative ionization mode using scheduled multiple reaction monitoring (MRM) coupled with the information-dependent acquisition (IDA) and an enhanced product ion scan (EPI). The scheduled MRM window was 90 s, and each LM parameter was optimized individually. LOX product analysis, using an MRM method with diagnostic ion fragments and identification, was performed as described previously.[922]

Determination of IL-6 and PGE2 in murine macrophages

Mouse macrophage cell line (J774A.1, ATCC® TIB-67™) was cultured in DMEM supplemented with FBS (ATCC® 30-2020™) to a final concentration of 10%. Cells were seeded in petri culture dishes (100 × 20 mm, Falcon®) at a density of 5×10^5 cells per dish and allowed to grow for 24 h. The medium was then replaced, and cells were treated with LPS (10 µg/ml) in the presence or absence of compounds **3, 6**, **7**, **9** (1 µM). Following incubations of 24 h, cells were collected with a cell scraper and, after centrifugation at 14000 × *g* for 10 min at 4 °C, the supernatant was collected and stored at −80°C for ELISA analysis[1023].

Animals

Male CD-1 mice (25–30 g of weight) were obtained from Charles River (Milan, Italy) and kept in an animal care facility under controlled temperature, humidity, with *ad libitum* access to water and standard laboratory chow diet. All experimental procedures were carried out according to the international and national law and policies (EU Directive 2010/63/EU for animal experiments, ARRIVE guidelines, and the Basel declaration including the 3R concept)[11,1224, 25]. All procedures were carried out to minimize the number of animals (n = 6 per group).

Induction of peritonitis in mice and Enzyme-Linked Immunosorbent Assay (ELISA)

To examine the anti-inflammatory action of compound **3**, we next evaluated the biological activity of compound **3** in a mouse model of zymosan-induced peritonitis, as as previously described.[13,14 26, 27] To this aim, mice were randomly divided into different experimental groups: control group (Ctrl), model group (zymosan + vehicle compound **3**), zymosan + compound **3** (0.1, 1, and 10 mg/kg), and zymosan + dexamethasone (3 mg/kg) group. Animals received the selected compound or dexamethasone intraperitoneally (i.p.) 30 min after i.p. injection of zymosan (500 mg/kg)[26, 27]. Ctrl and model groups received an equal volume of vehicle (PBS or DMSO/saline 1:3, respectively). Peritoneal exudates were collected at selected time points (4 and 24 h) by washing the cavity with 2 mL of PBS. Then cell number of lavage fluids was determined by TC10 automated cell counter (Bio-Rad, Milan, Italy) using disposable slides. The remaining lavage fluids were centrifuged at 3000 rpm for 20 min at 4 °C, and supernatants were frozen at −80 °C for further ELISA analysis according to the manufacturer instructions (eBioscience Co., San Diego, CA, USA)[10 23] of IL-1β, IL-6, PGE_2 and IL-10 following the procedure previously described[15-17 28-30]. Dexamethasone and zymosan A were purchased from Sigma-Aldrich (Milan, Italy). DMSO was purchased from Merck (Italy). Unless otherwise stated, all the other reagents were purchased from Carlo Erba (Milan, Italy).

Statistical analysis

The data and statistical analysis in this study comply with the international recommendations on experimental design and analysis in preclinical pharmacology[18-2031-33]. The results obtained were expressed as the mean ± S.D. or as mean ± S.E.M., as reported in detail in the figure legends. IC_{50} values were calculated by nonlinear regression using GraphPad Prism Version 8.0 software (San Diego, CA) one site binding competition. Statistical evaluation of the data was performed by one-

way ANOVA followed by a Bonferroni post hoc test for multiple comparison. GraphPad Prism 8.0 software (San Diego, CA, USA) was used for analysis.

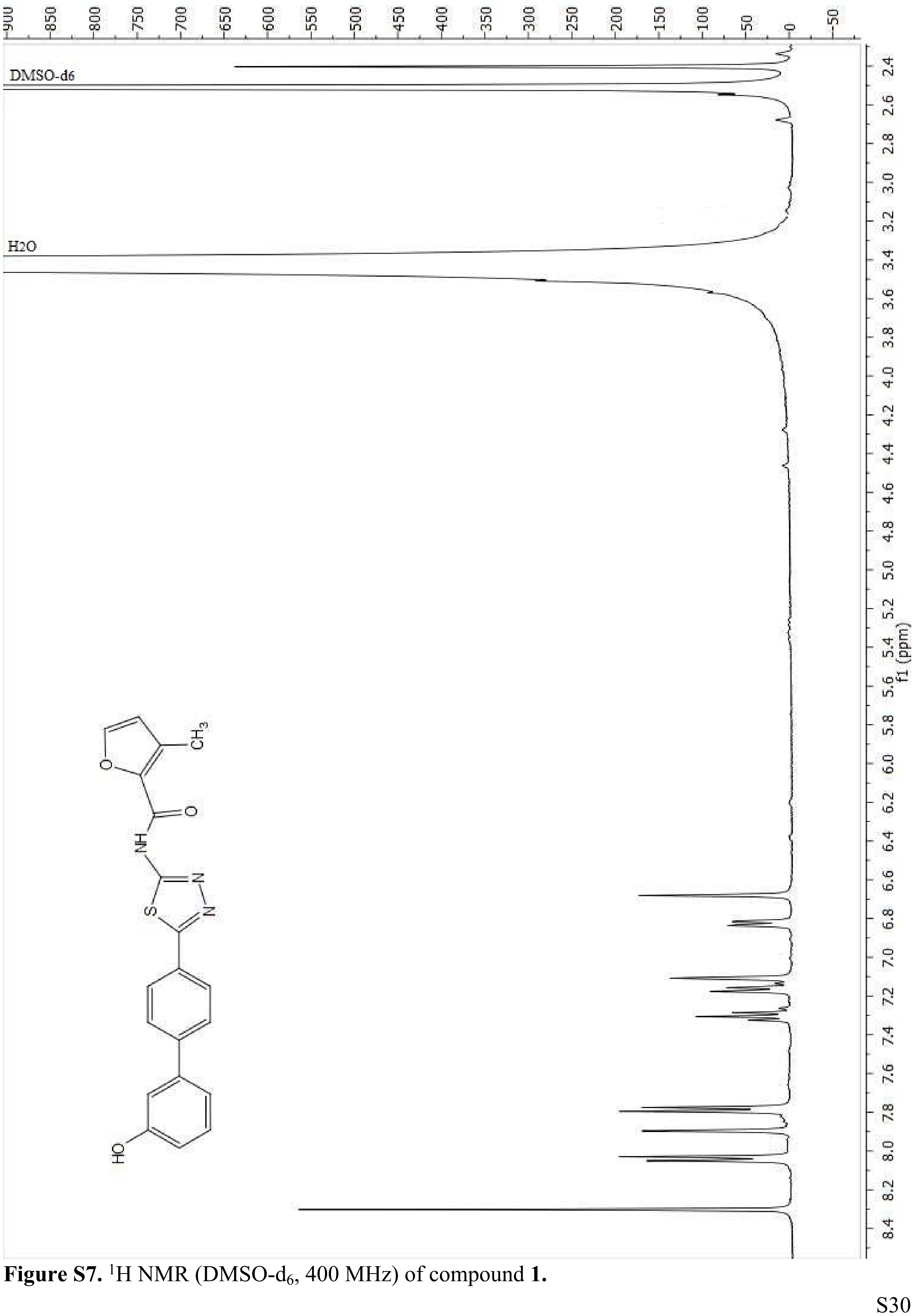

Figure S7. ^{1}H NMR (DMSO-d$_6$, 400 MHz) of compound **1.**

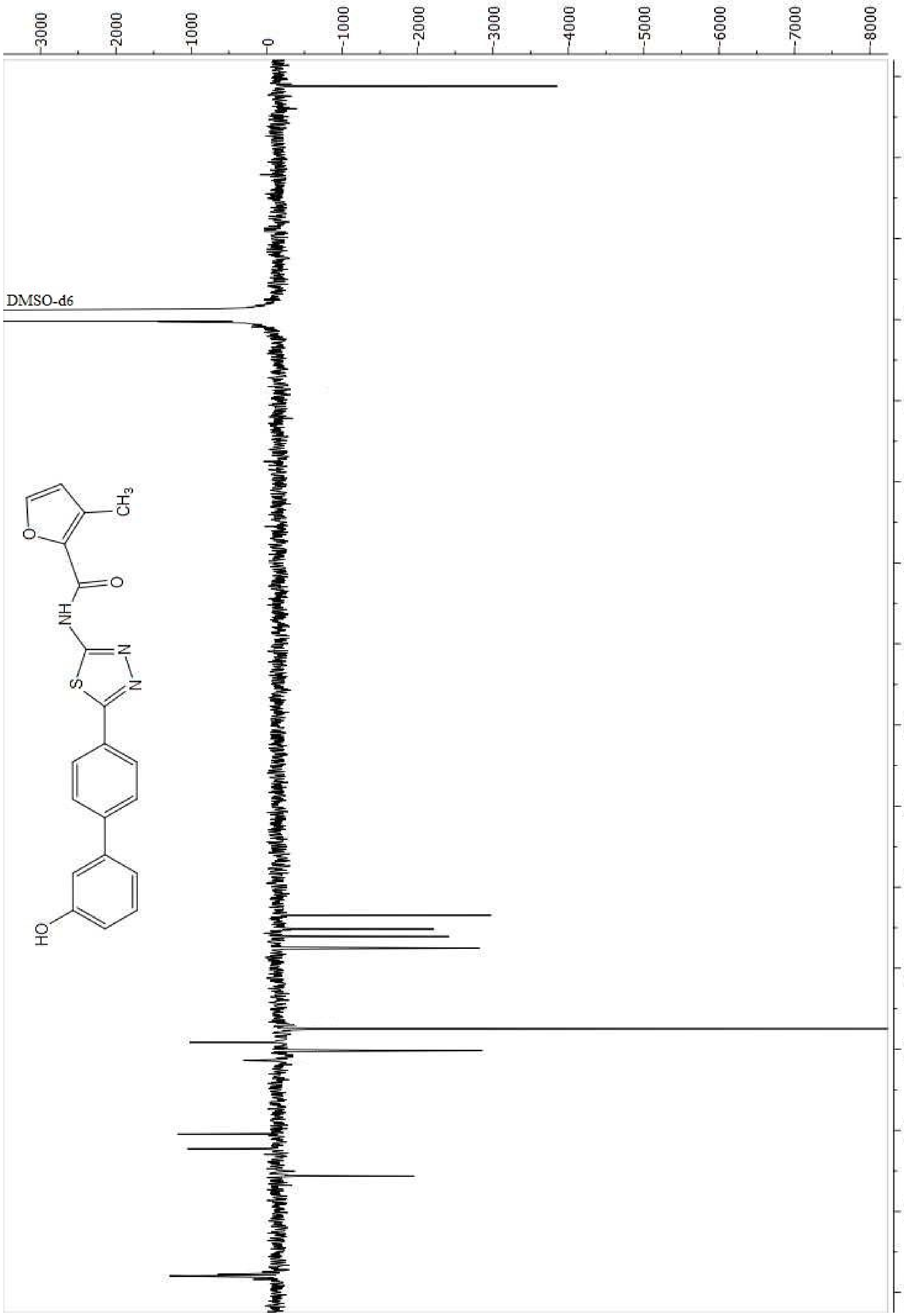

Figure S8. DEPTQ NMR (DMSO-d_6, 100 MHz) of compound **1.**

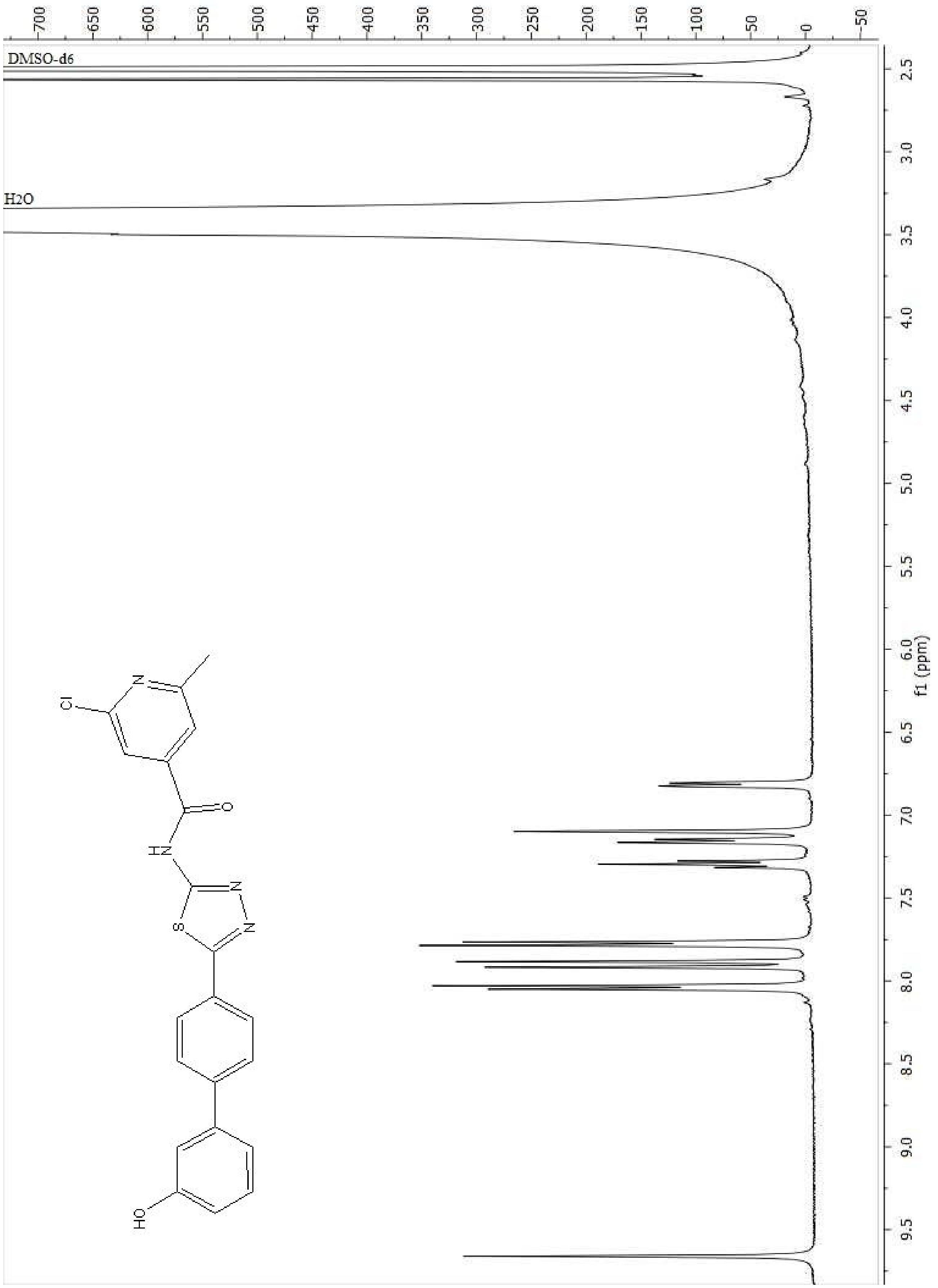

Figure S9. ^{1}H NMR (DMSO-d$_6$, 400 MHz) of compound **2**.

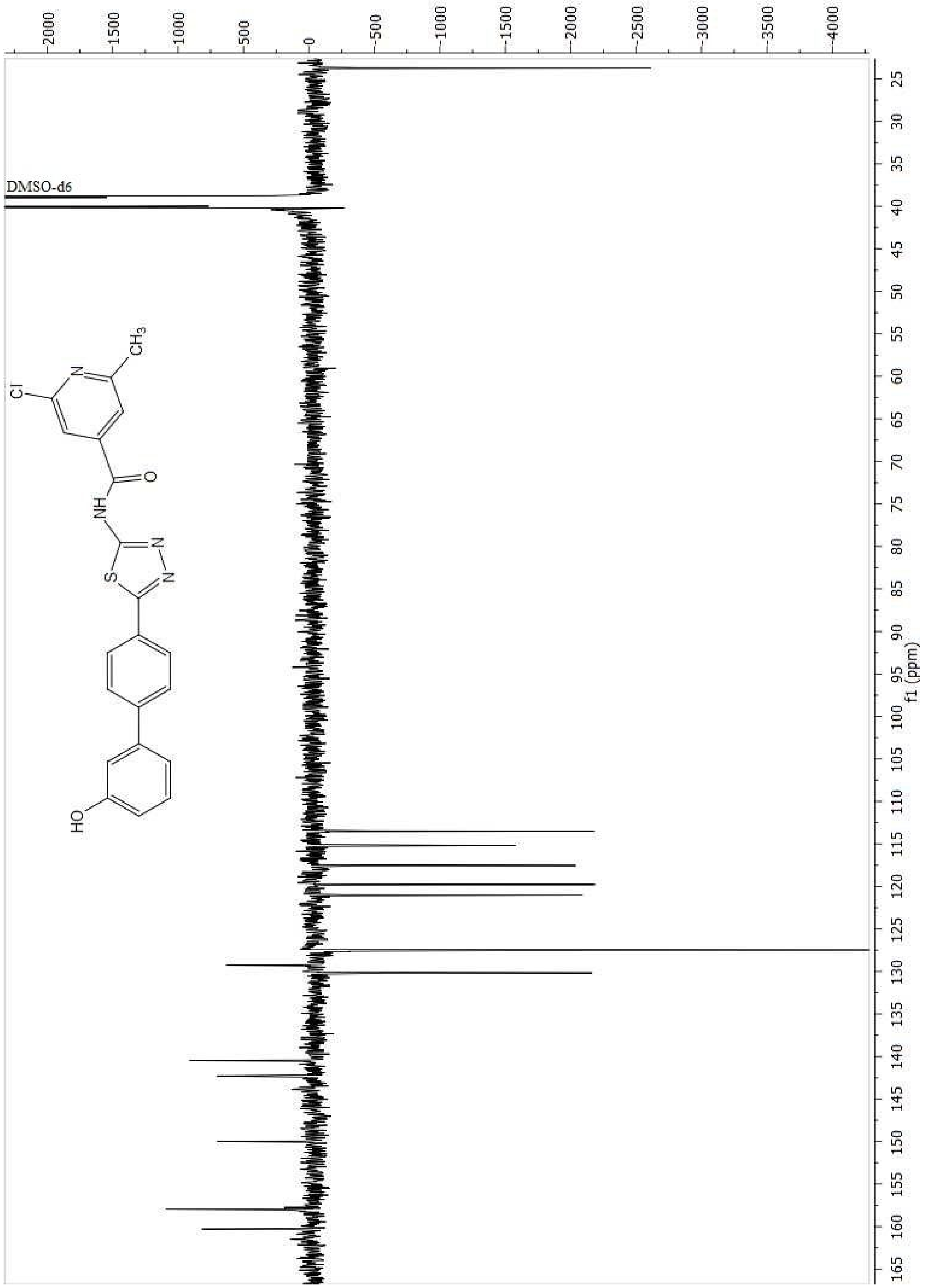

Figure S10. DEPTQ NMR (DMSO-d_6, 100 MHz) of compound **2**.

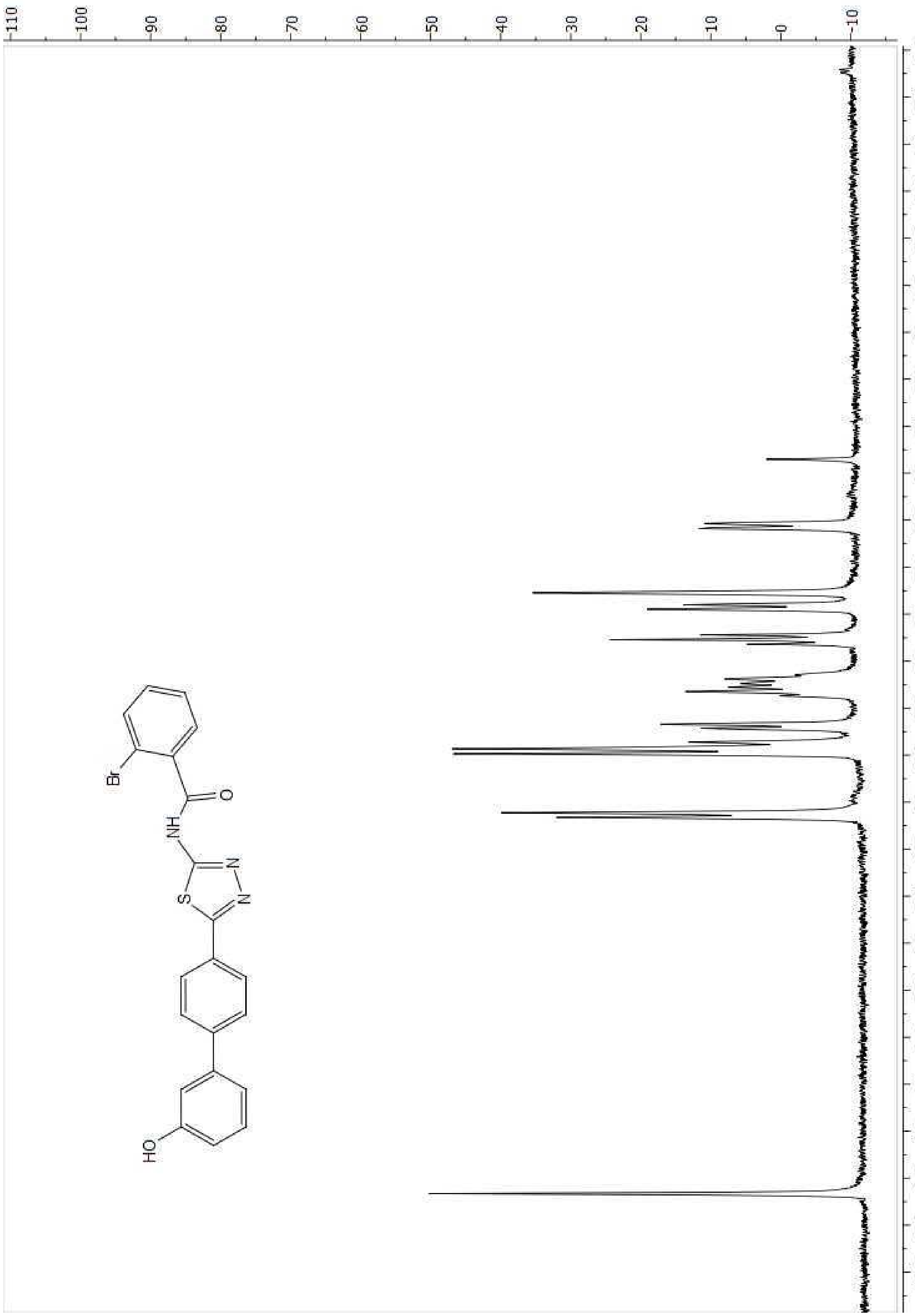

Figure S11. ^{1}H NMR (DMSO-d_6, 400 MHz) of compound **3**.

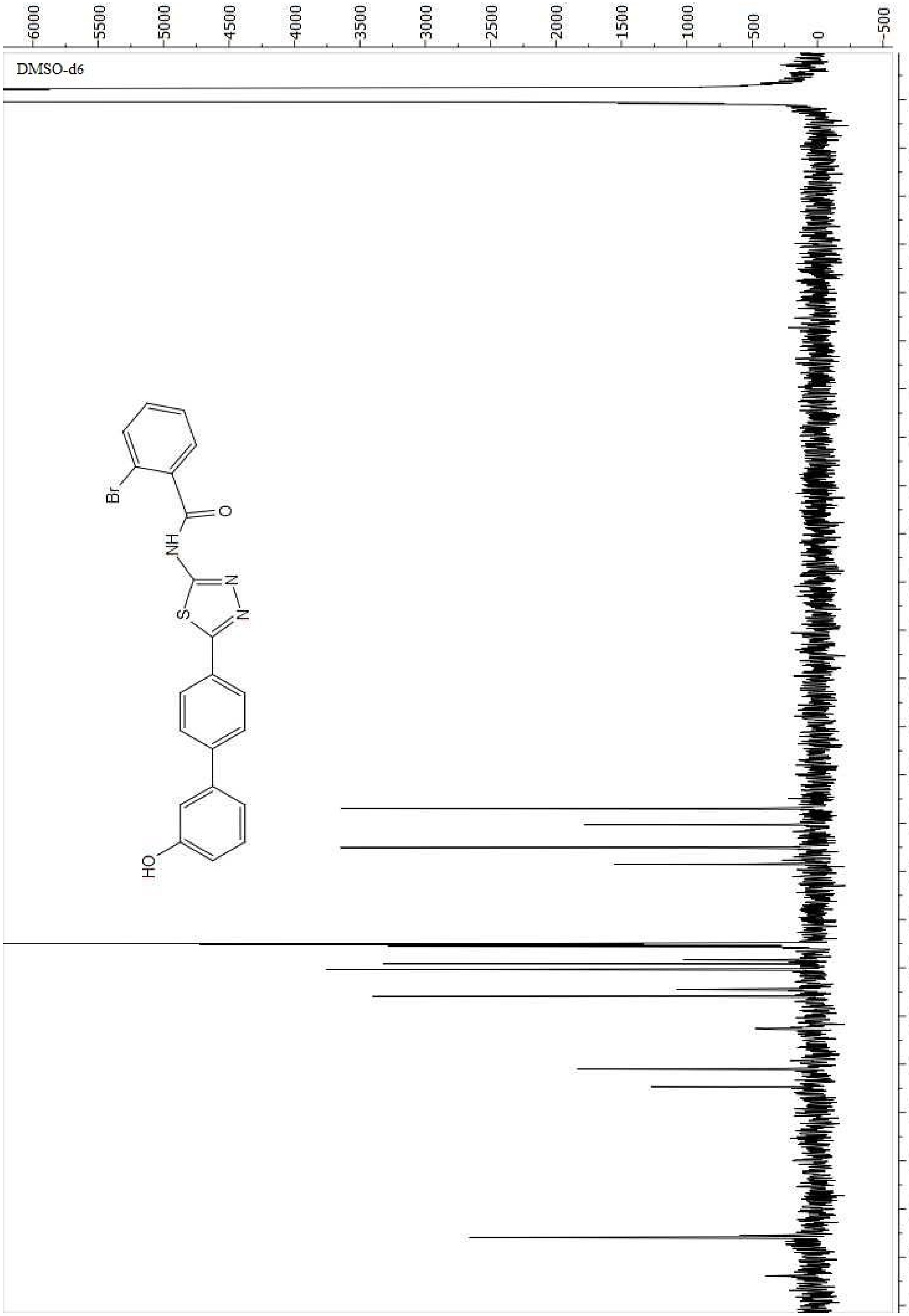

Figure S12. ^{13}C NMR (DMSO-d_6, 100 MHz) of compound **3**.

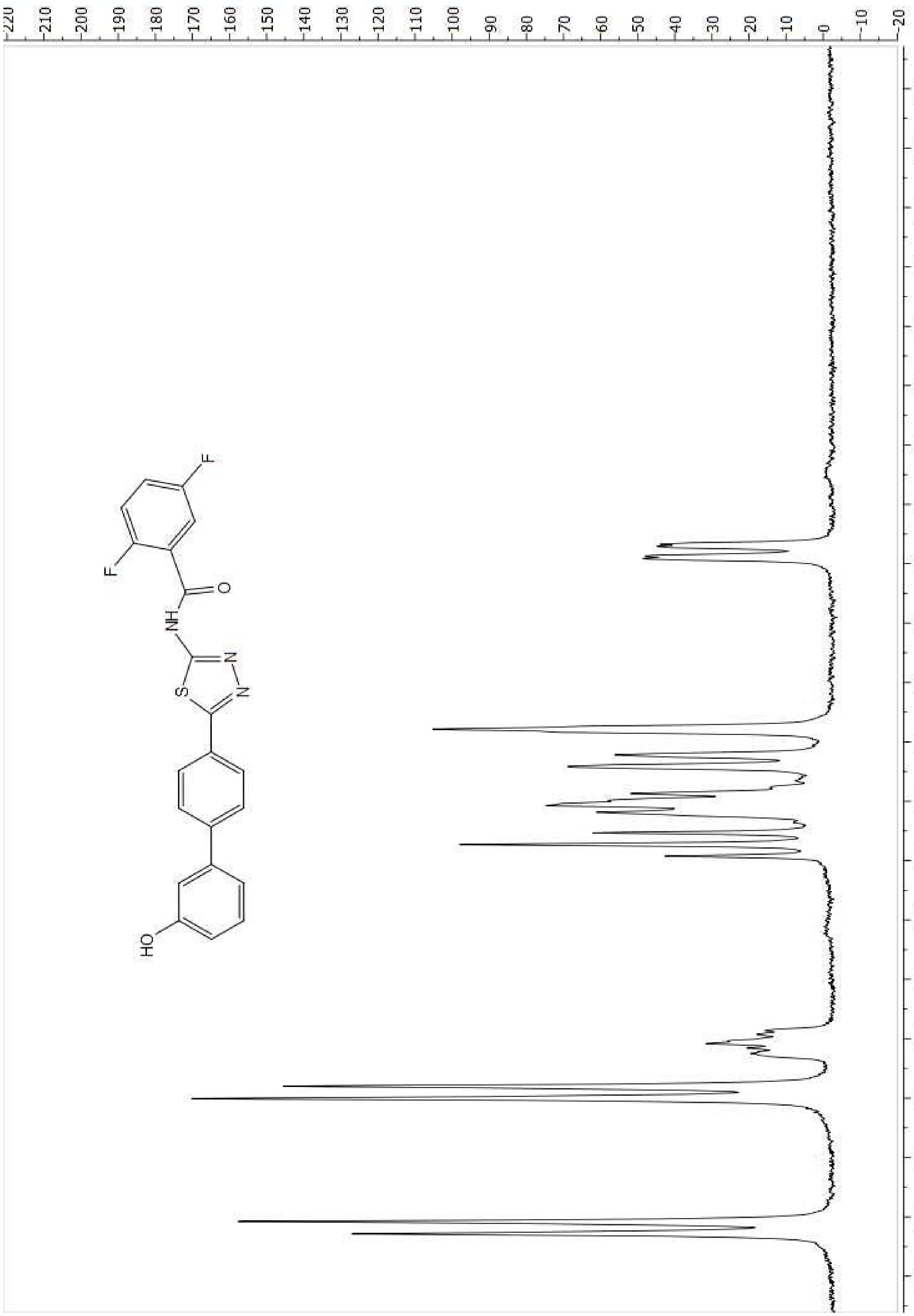

Figure S13. ^{1}H NMR (DMSO-d_6, 400 MHz) of compound **4**.

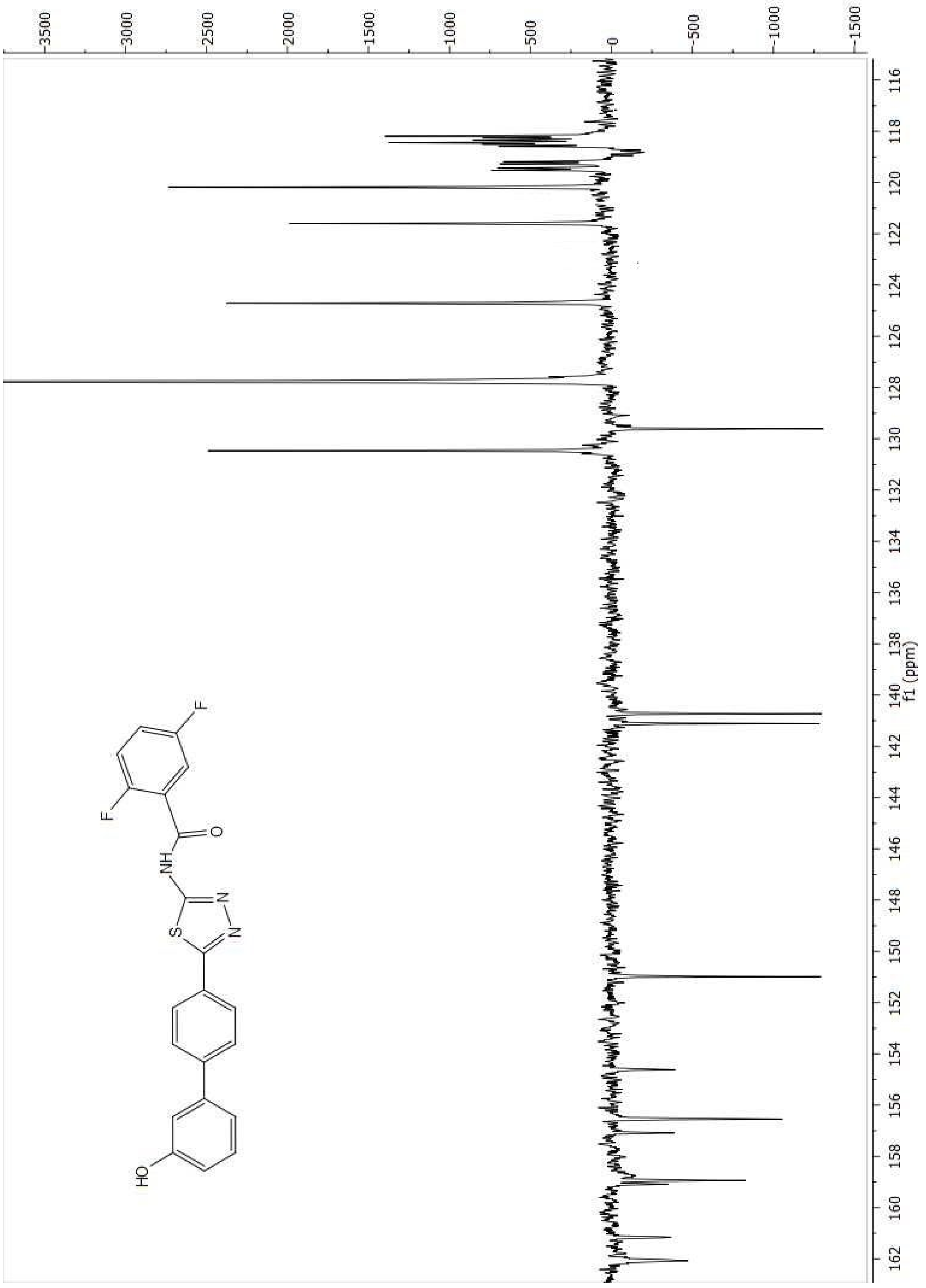

Figure S14. DEPTQ NMR (DMSO-d_6, 100 MHz) of compound **4**.

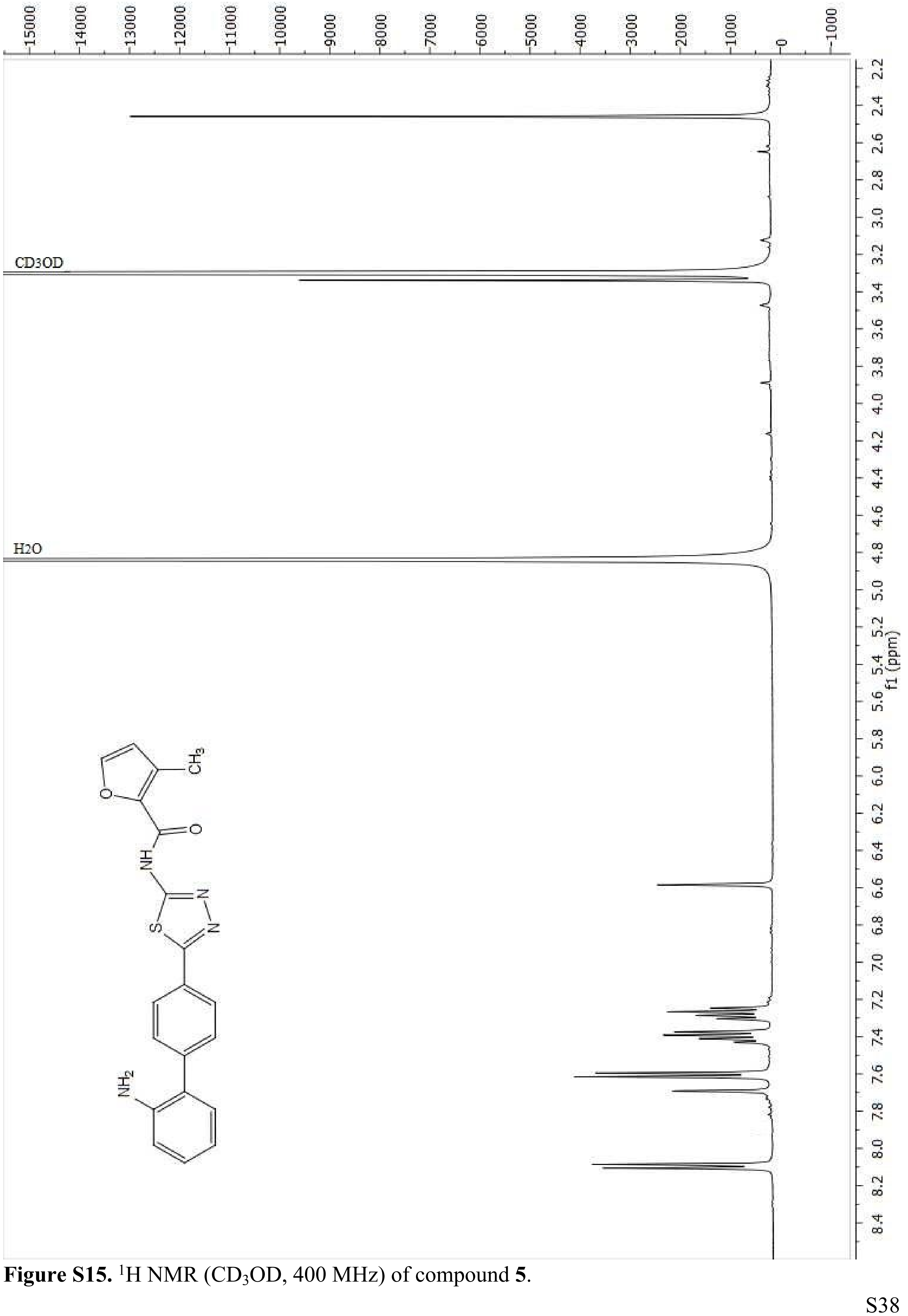

Figure S15. ^{1}H NMR (CD_3OD, 400 MHz) of compound **5**.

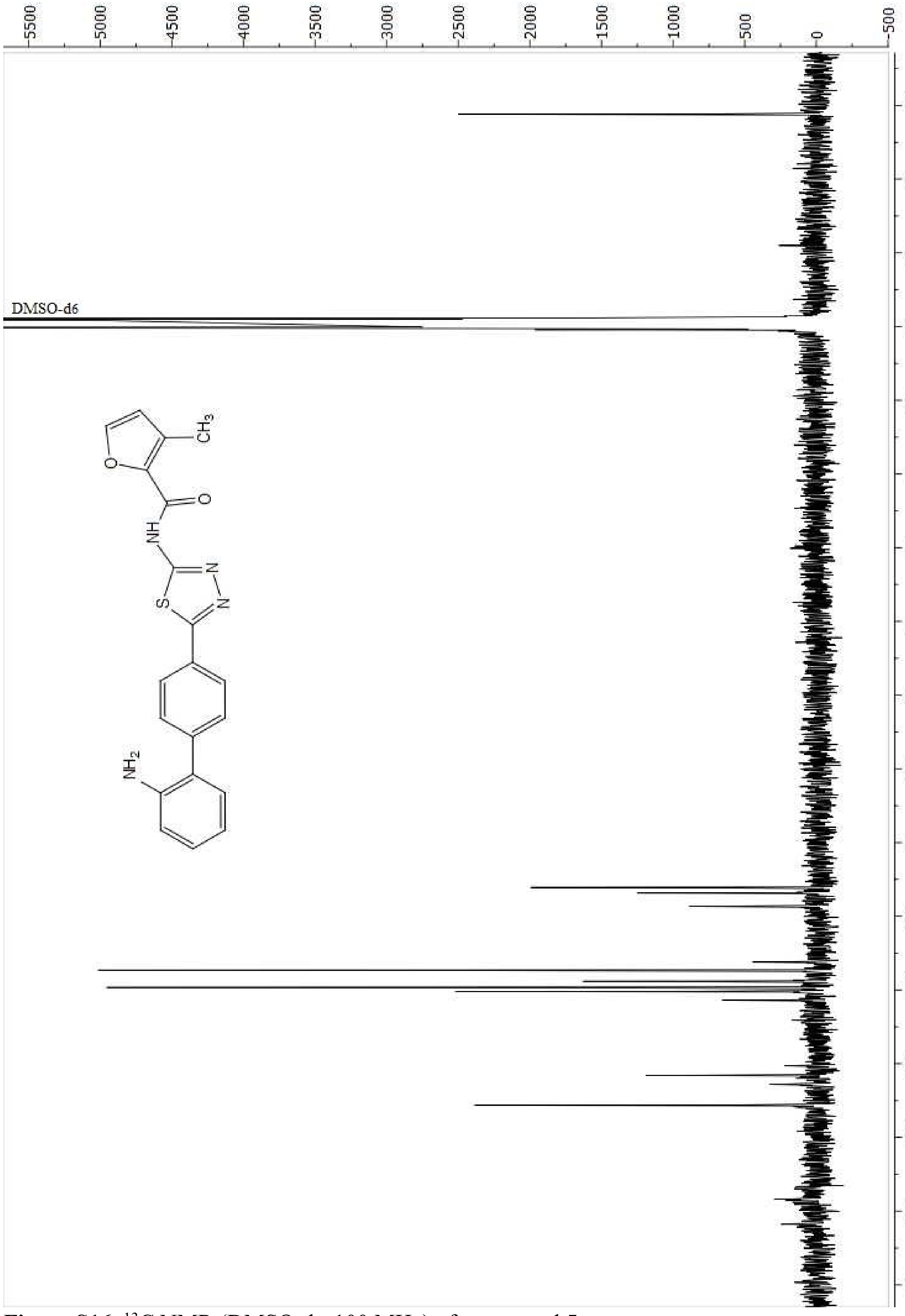

Figure S16. ^{13}C NMR (DMSO-d_6, 100 MHz) of compound **5**.

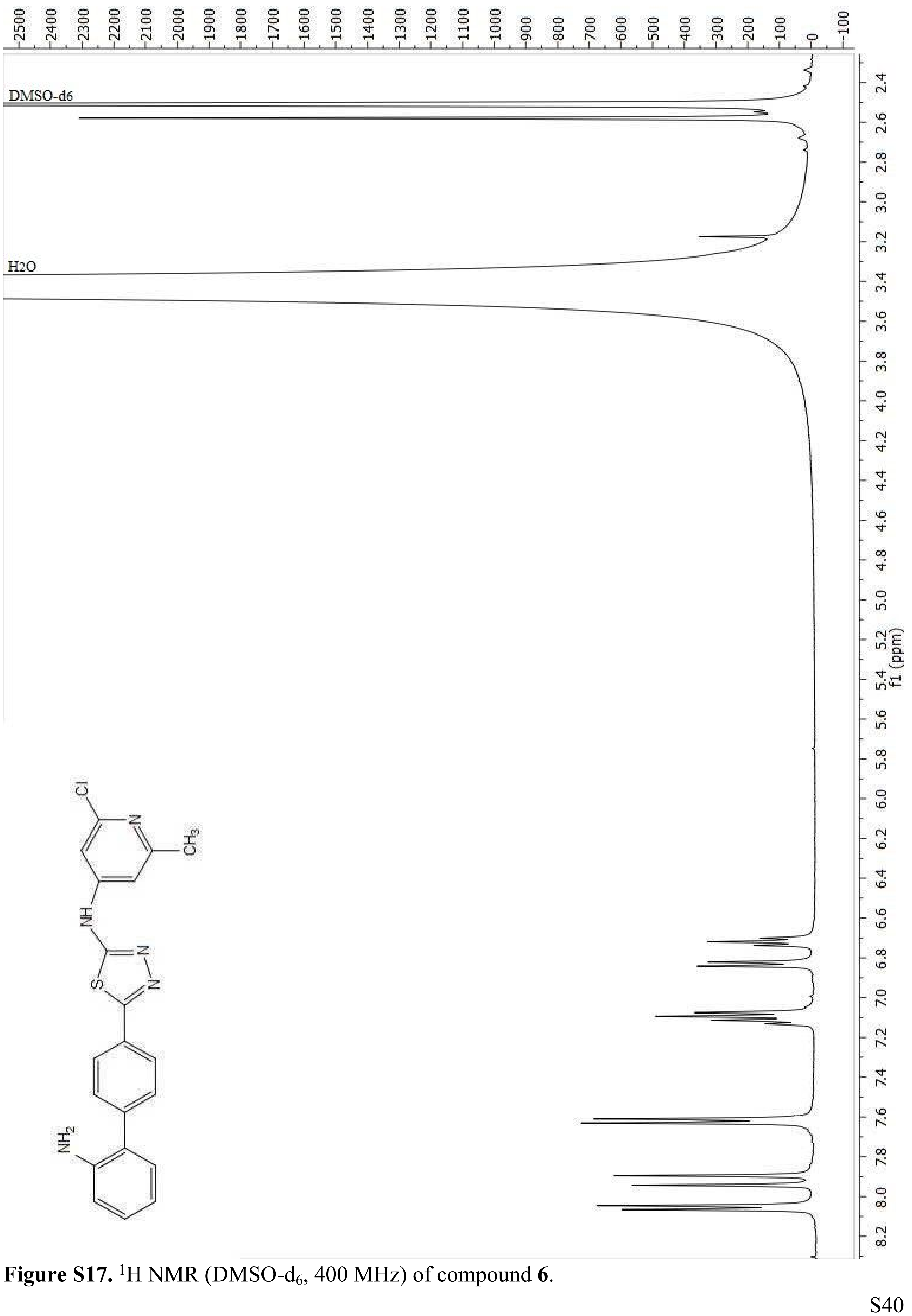

Figure S17. ^{1}H NMR (DMSO-d_6, 400 MHz) of compound **6**.

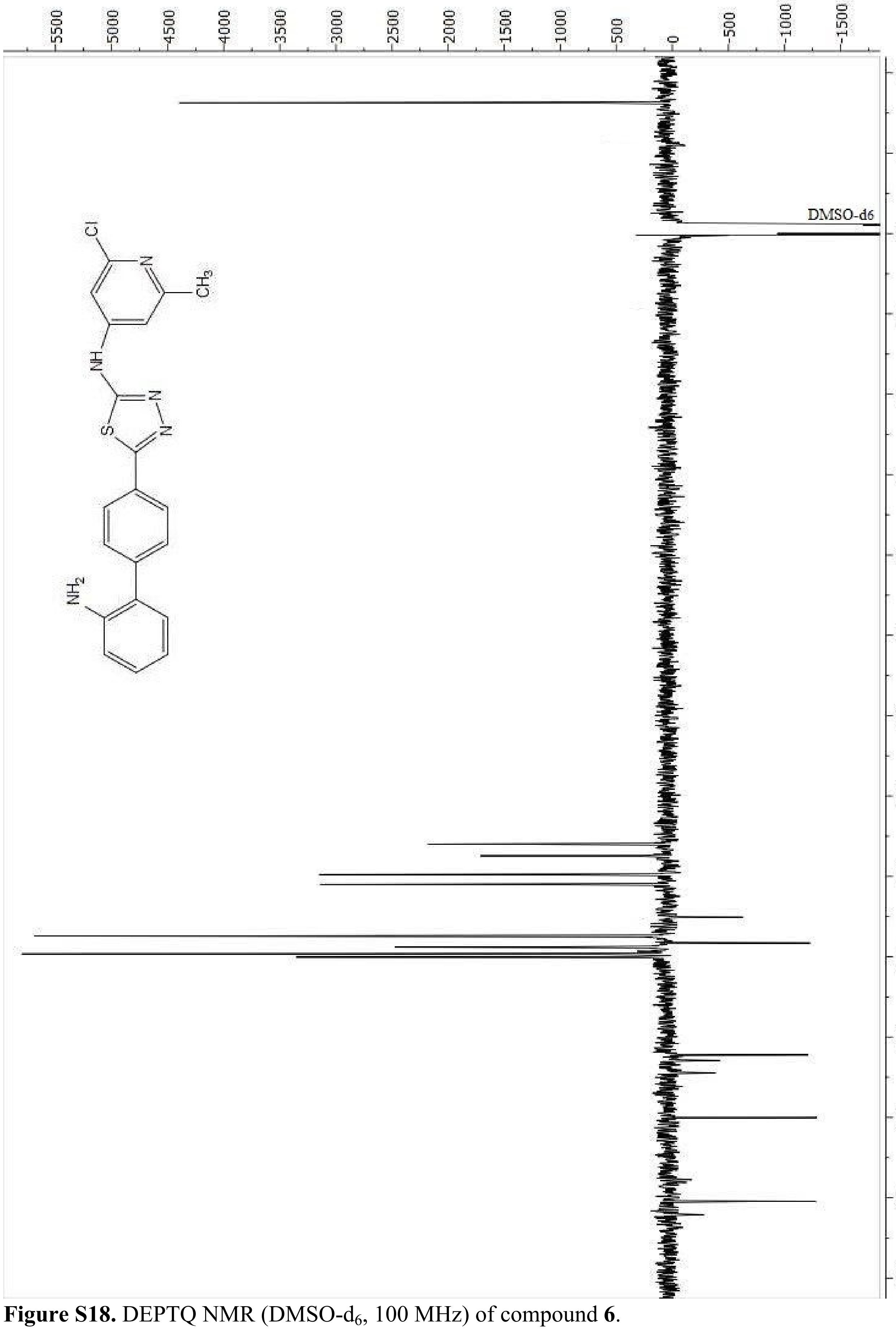

Figure S18. DEPTQ NMR (DMSO-d$_6$, 100 MHz) of compound **6**.

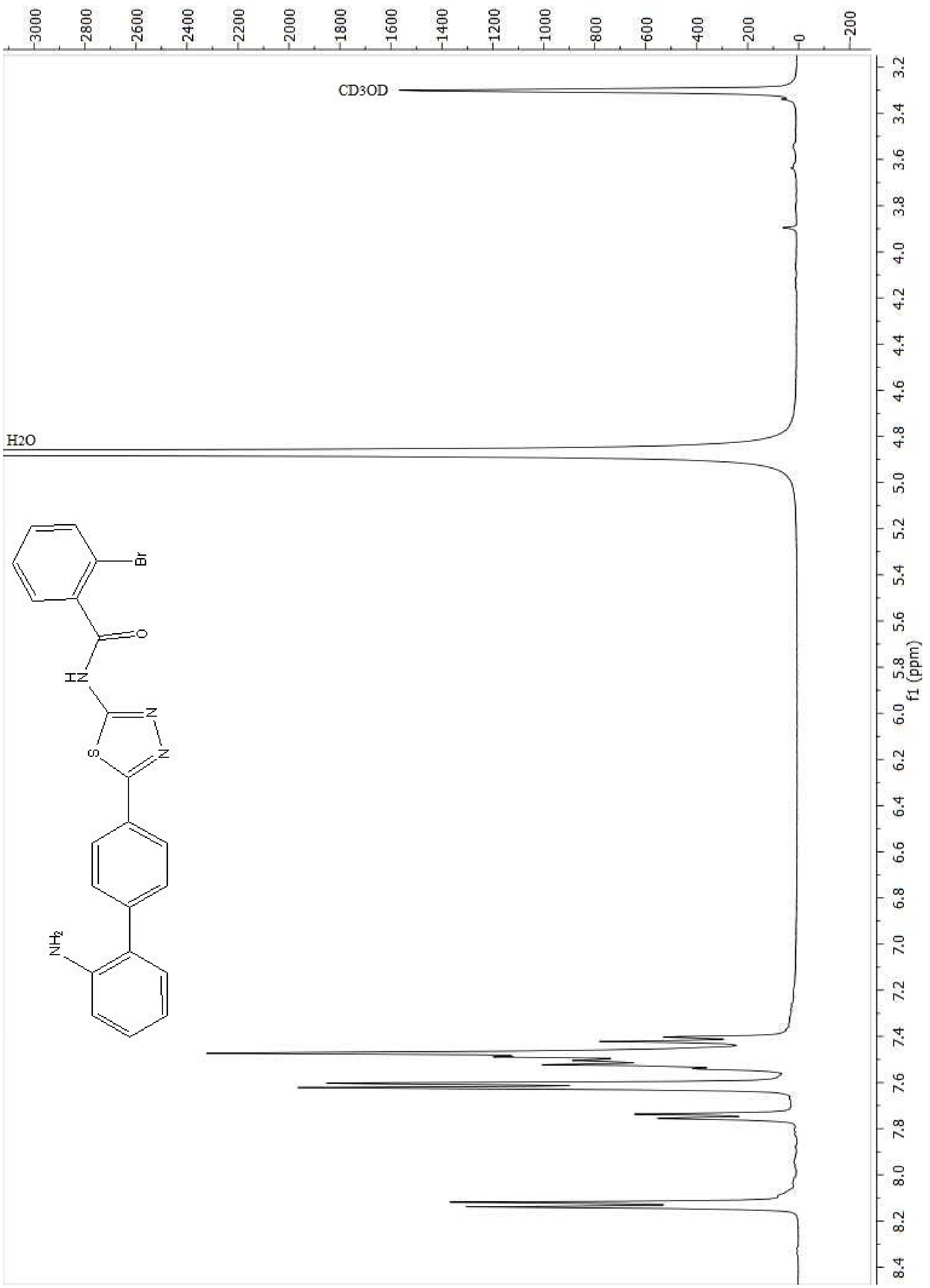

Figure S19. ^{1}H NMR (CD_3OD, 400 MHz) of compound **7**.

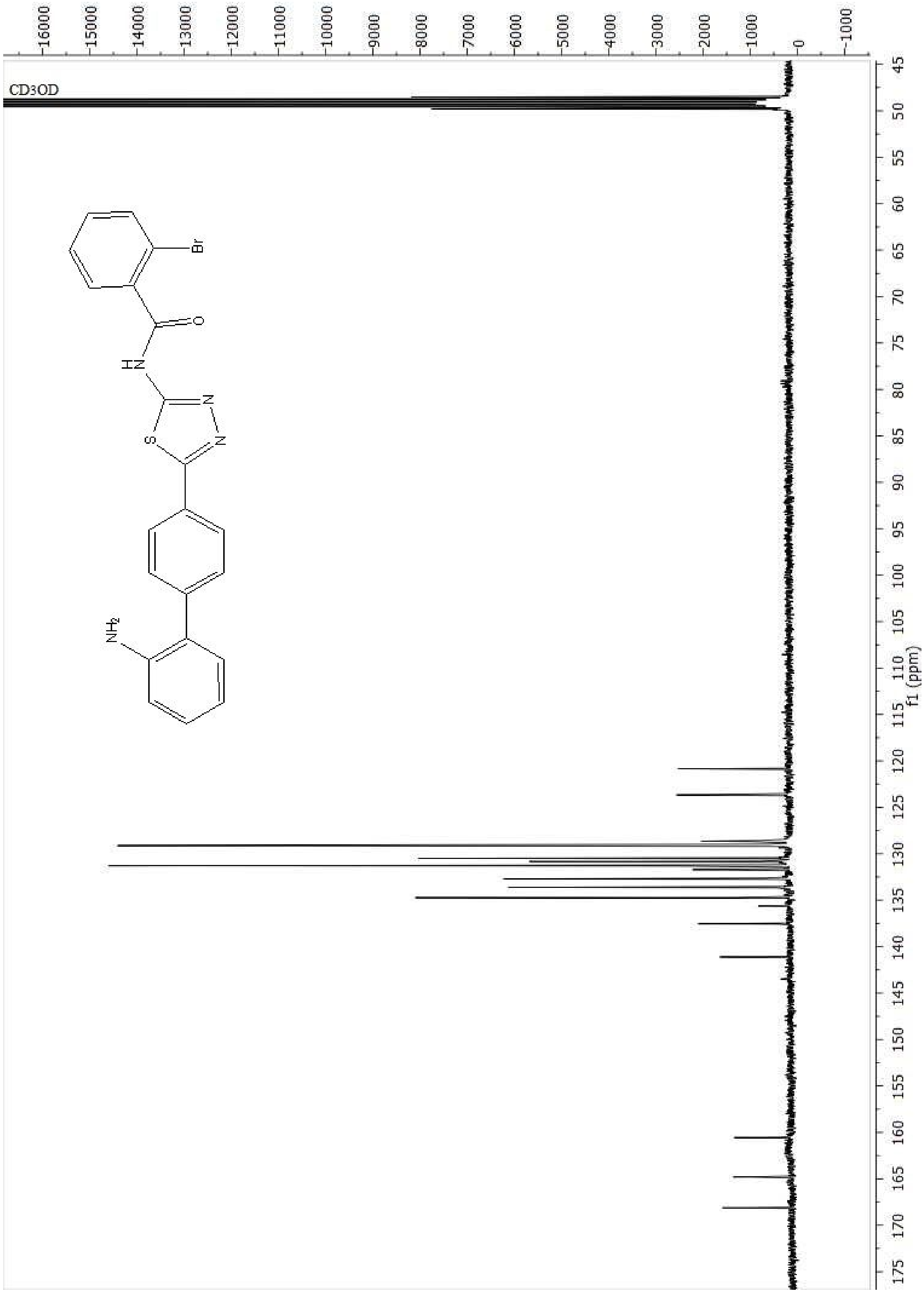

Figure S20. ^{13}C NMR (CD_3OD, 100 MHz) of compound **7**.

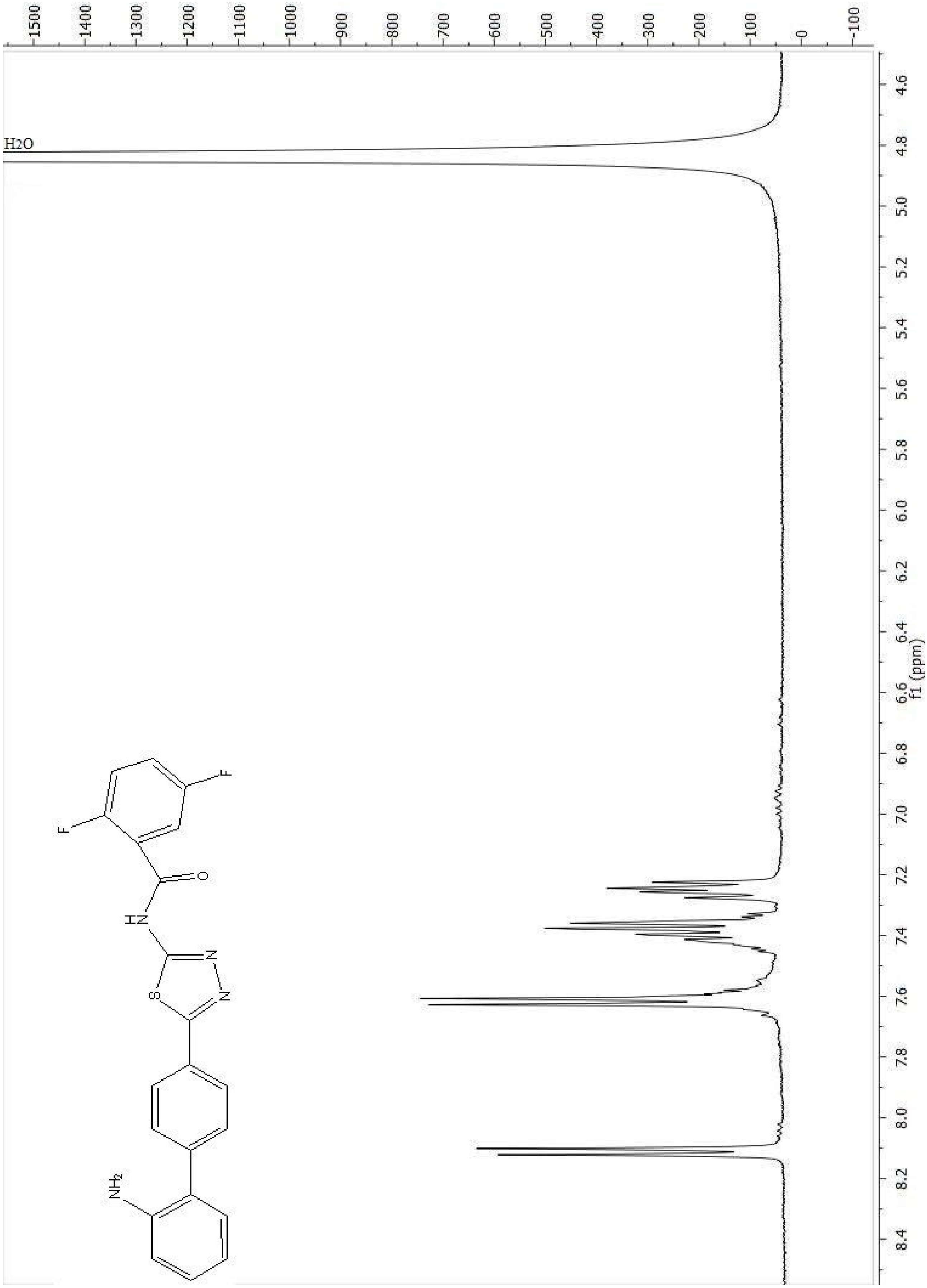

Figure S21. ^{1}H NMR (CD_3OD, 400 MHz) of compound **8.**

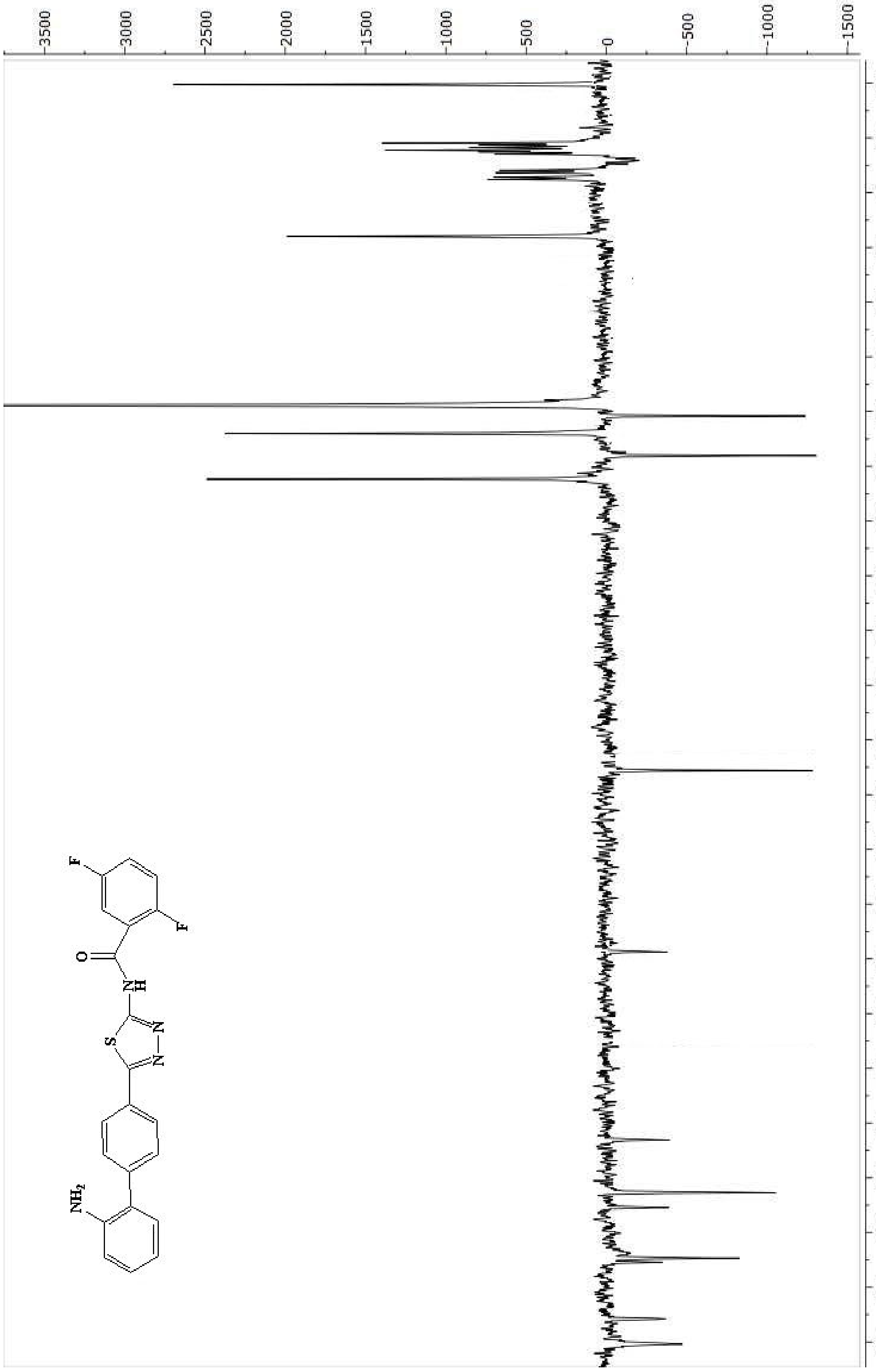

Figure S22. DEPTQ NMR (DMSO-d$_6$, 100 MHz) of compound **8**.

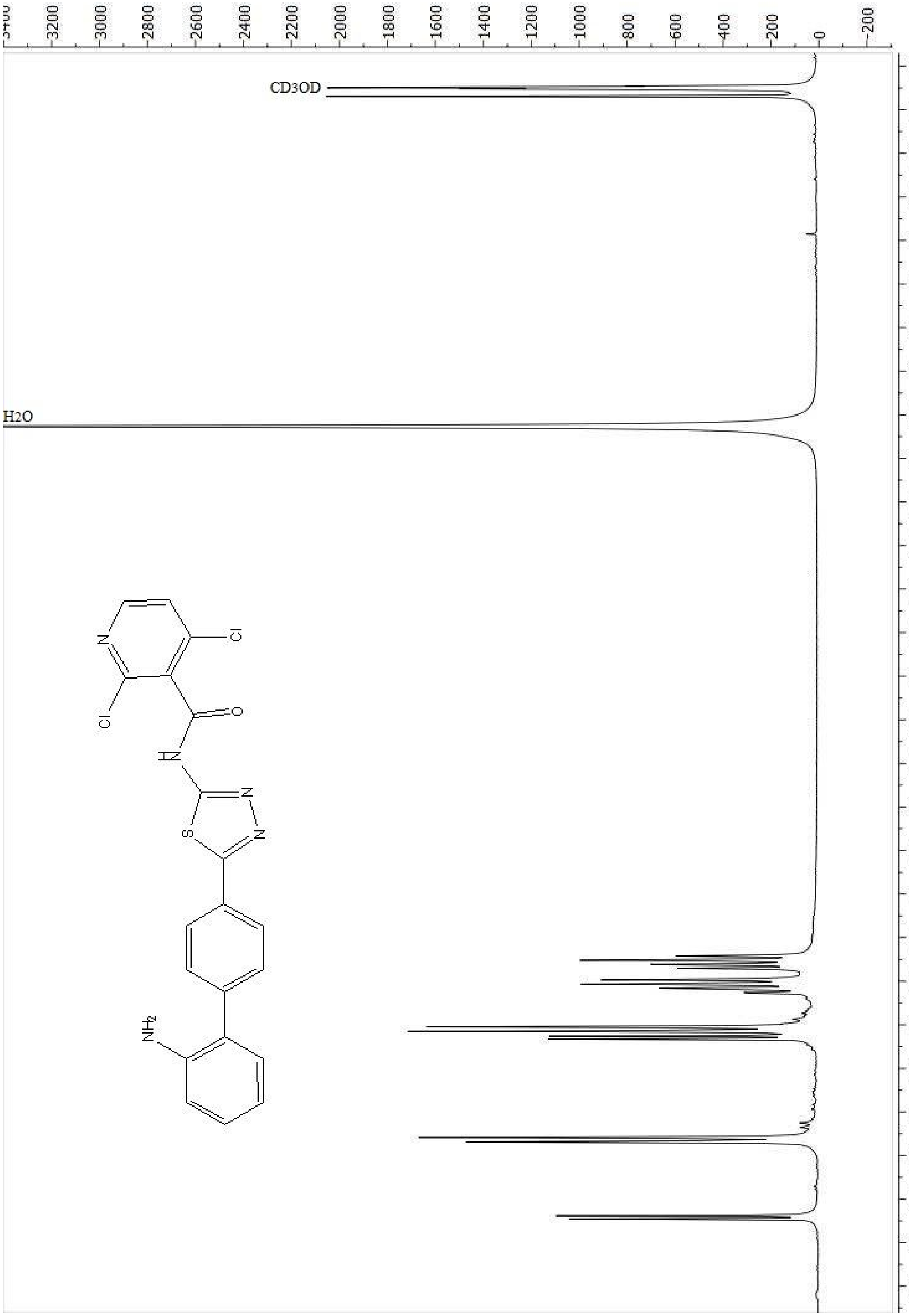

Figure S23. ^{1}H NMR (CD_3OD, 400 MHz) of compound **9**.

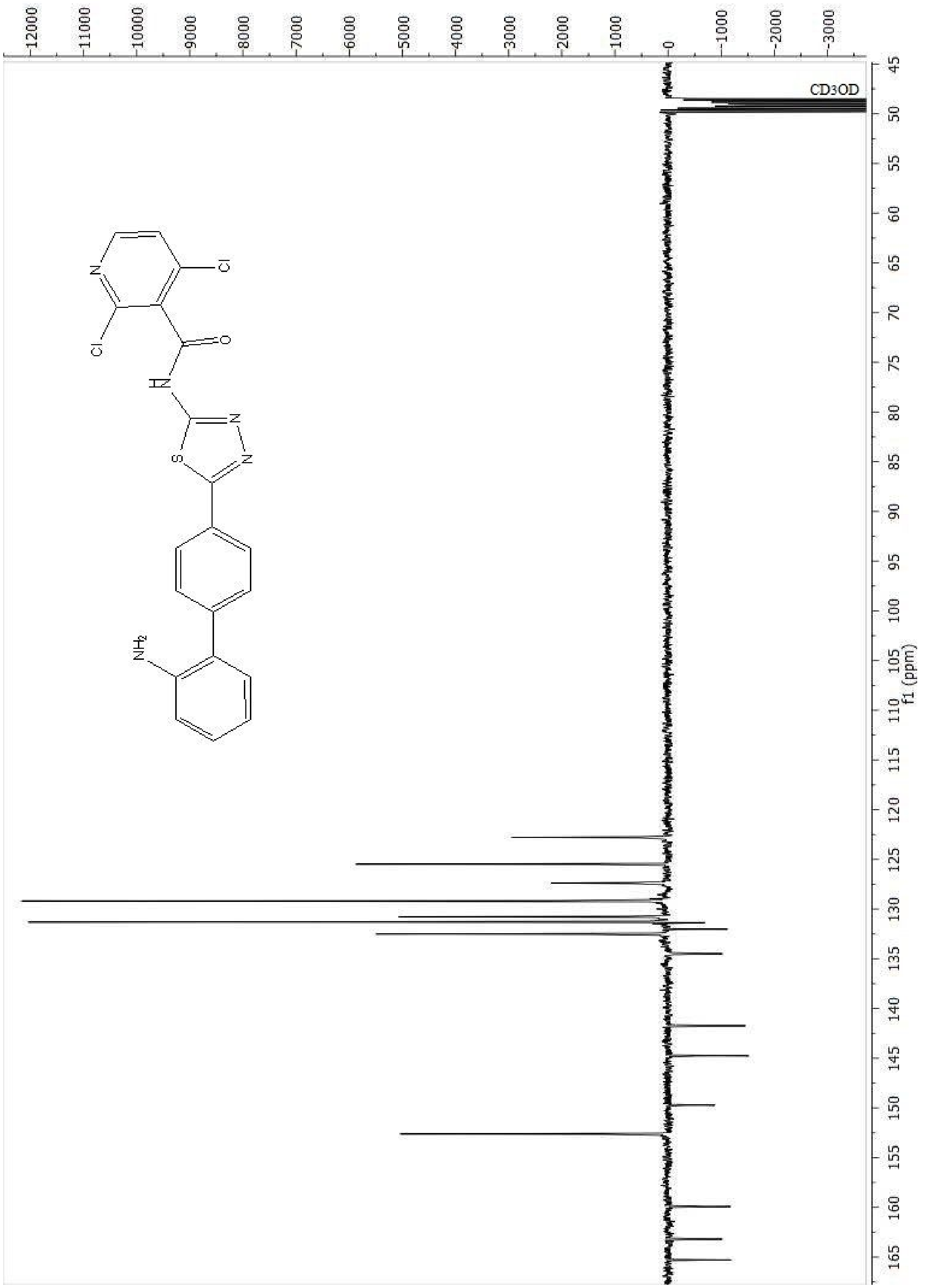

Figure S24. DEPTQ NMR (CD_3OD, 100 MHz) of compound **9**.

HR MS spectra of compounds 3, 6, 7, 9

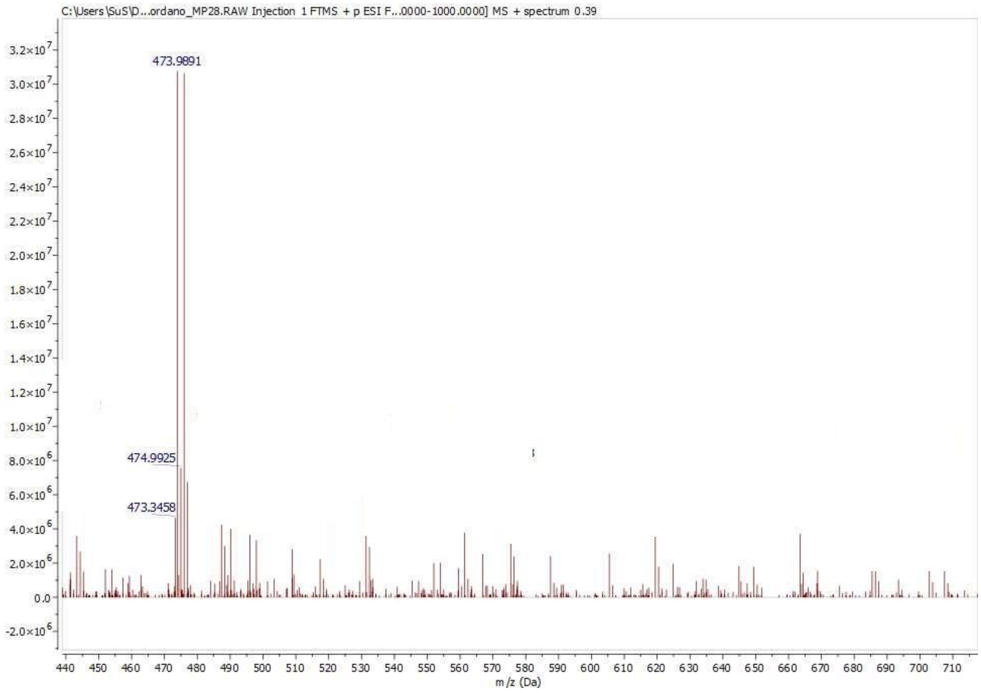

Figure S25. HRMS spectrum of compound **3.**

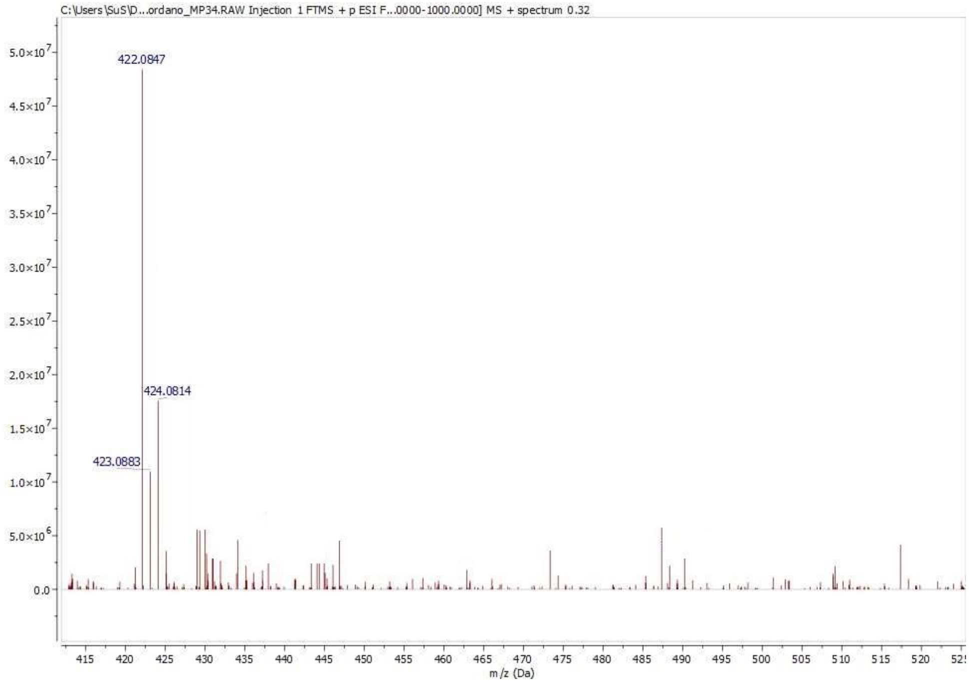

Figure S26. HRMS spectrum of compound **6**.

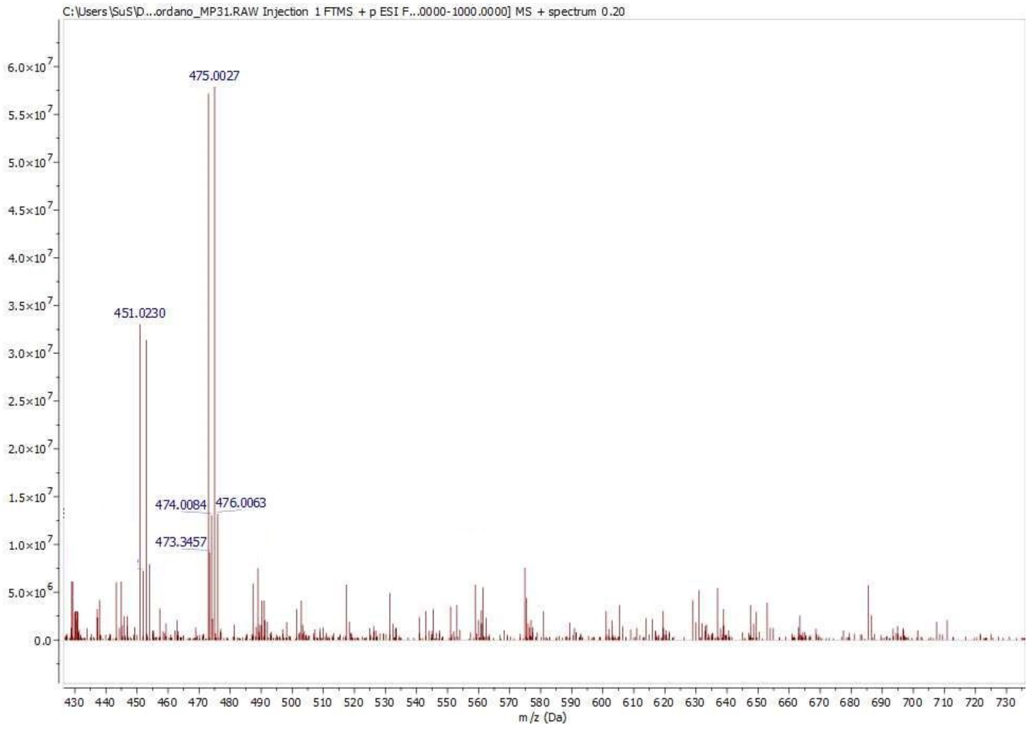

Figure S27. HRMS spectrum of compound **7**.

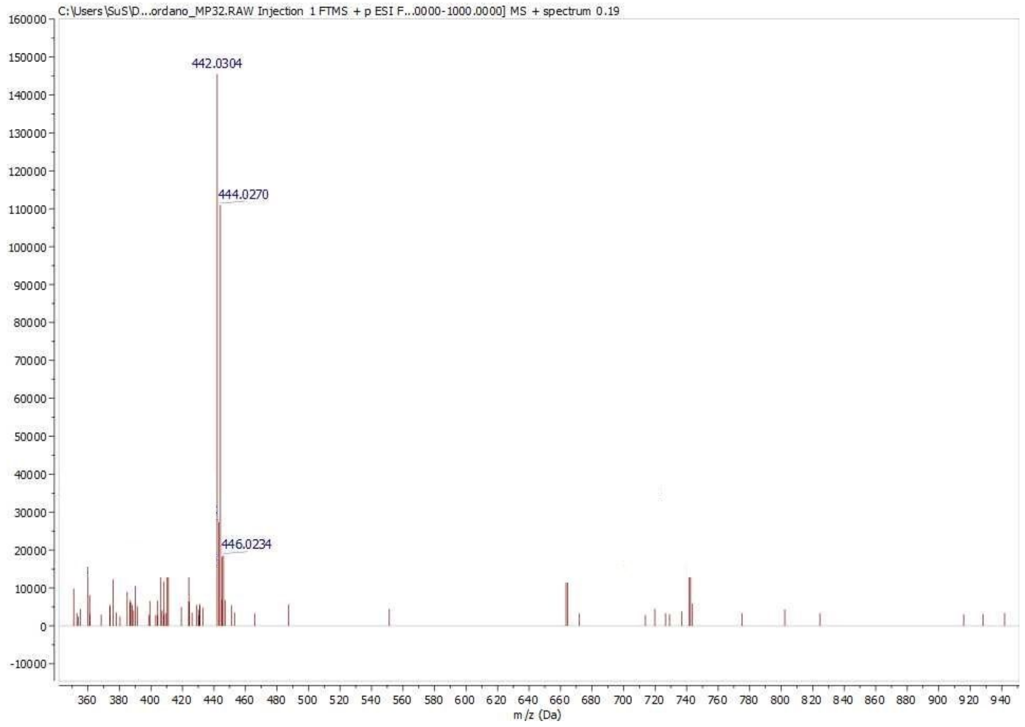

Figure S28. HR MS spectrum of compound **9**.

HPLC method for analysis of compounds 3, 6, 7, and 9.

A Synergi Fusion Phenomenex® C18 reversed-phase column (250 x 4.60mm, 4μ, 80 Å) ,was used at a flow rate = 1 mL/min, with the following gradient: from 5% B to 100% B in 50 min.
The binary solvent system (A/B) was as follows: 0.1% TFA in water (A) and 0.1% TFA in CH_3CN (B); the absorbance was detected at 280 nm.

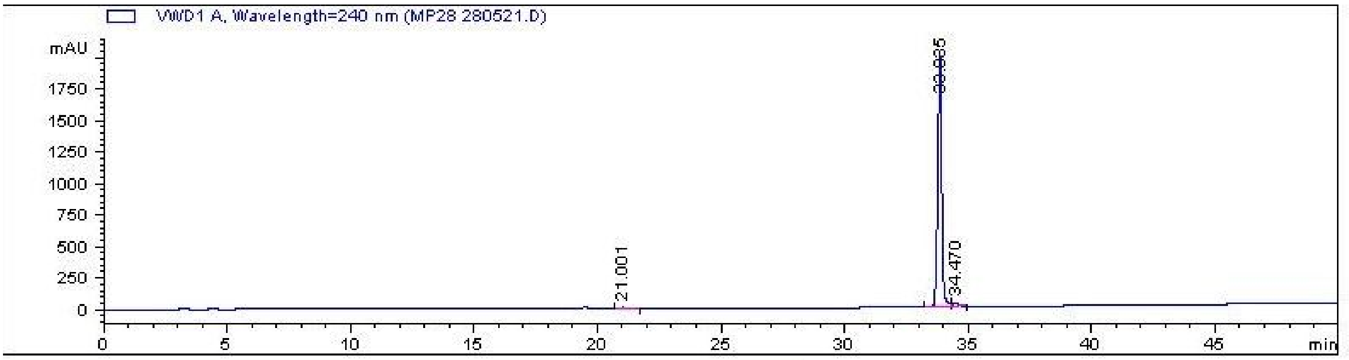

#	Time	Area	Height	Width	Area%	Symmetry
1	21.001	121.7	7.8	0.2172	0.510	1.226
2	33.835	23153.7	2031.4	0.173	97.024	1.138
3	34.47	588.6	34.4	0.2396	2.466	0.653

Figure S29. HPLC chromatogram of compound **3**.

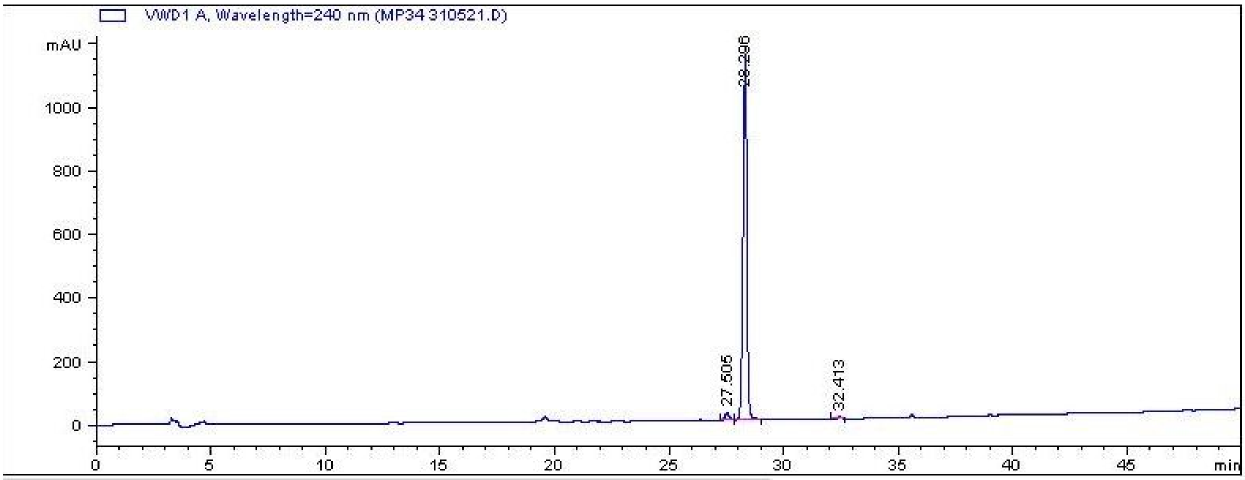

#	Time	Area	Height	Width	Area%	Symmetry
1	27.505	358.9	26.2	0.1999	2.744	0.783
2	28.296	12617.5	1149.6	0.1641	96.458	1.318
3	32.413	104.4	9.3	0.1704	0.798	1.131

Figure S30. HPLC chromatogram of compound **6**.

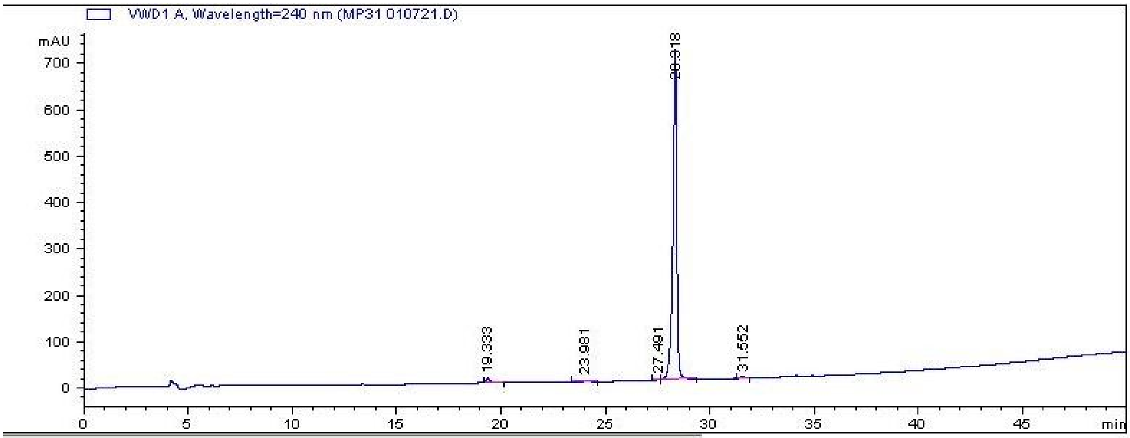

#	Time	Area	Height	Width	Area%	Symmetry
1	19.333	152.2	9.9	0.2105	1.670	0.468
2	23.981	55.7	2.9	0.2618	0.611	0.425
3	27.491	36	3.8	0.1424	0.395	1.092
4	28.318	8828.2	712.6	0.1755	96.834	1.509
5	31.552	44.7	4.1	0.1617	0.491	0.838

Figure S31. HPLC chromatogram of compound **7**.

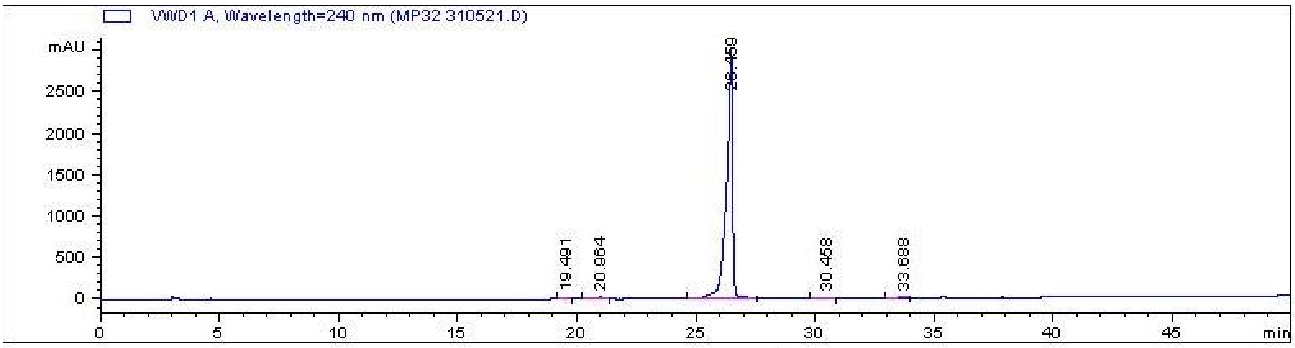

#	Time	Area	Height	Width	Area%	Symmetry
1	19.491	234.9	16.7	0.1998	0.426	0.721
2	20.964	351.6	21.4	0.2236	0.638	2.17
3	26.459	54151.4	2996.4	0.2428	98.295	2.857
4	30.458	149	8.1	0.2476	0.270	1.509
5	33.688	203.6	10.1	0.2708	0.370	2.761

Figure S32. HPLC chromatogram of compound **9**.

References

(1) Schrödinger, Release 2021-2: LigPrep, Schrödinger, LLC, New York, NY, **2021**.
(2) Schrödinger, Release 2021-2: QikProp, Schrödinger, LLC, New York, NY, **2021**.
(3) Schrödinger, Release 2021-2: VSW, Schrödinger, LLC, New York, NY, **2021**.
(4) Daina, A.; Michielin, O.; Zoete, V. SwissADME: a free web tool to evaluate pharmacokinetics, drug-likeness and medicinal chemistry friendliness of small molecules. *Sci. Rep.* **2017**, *7*, 42717.
(5) The Universal Protein Resource (UniProt). *Nucl. Acids Res.* **2007**, *36*, D190-D195.
(6) Jacobson, M. P.; Pincus, D. L.; Rapp, C. S.; Day, T. J. F.; Honig, B.; Shaw, D. E.; Friesner, R. A. A hierarchical approach to all-atom protein loop prediction. P*roteins: Struct. Funct. Bio* **2004**, *55*, 351-367.
(7) Jacobson, M. P.; Friesner, R. A.; Xiang, Z.; Honig, B. On the Role of the Crystal Environment in Determining Protein Side-chain Conformations. *J. Mol. Biol.* **2002**, *320*, 597-608.
(8) Schrödinger, Release 2021-2: Prime, Schrödinger, LLC, New York, NY, **2021**.
(9) Morris, A. L.; MacArthur, M. W.; Hutchinson, E. G.; Thornton, J. M. Stereochemical quality of protein structure coordinates. *Proteins: Struct. Funct. Genet.* **1992**, *12*, 345-364.
(10) Friesner, R. A.; Banks, J. L.; Murphy, R. B.; Halgren, T. A.; Klicic, J. J.; Mainz, D. T.; Repasky, M. P.; Knoll, E. H.; Shelley, M.; Perry, J. K.; Shaw, D. E.; Francis, P.; Shenkin, P. S. Glide: a new approach for rapid, accurate docking and scoring. 1. Method and assessment of docking accuracy. *J. Med. Chem.* **2004**, *47*, 1739-1749.
(11) Friesner, R. A.; Murphy, R. B.; Repasky, M. P.; Frye, L. L.; Greenwood, J. R.; Halgren, T. A.; Sanschagrin, P. C.; Mainz, D. T. Extra precision glide: docking and scoring incorporating a model of hydrophobic enclosure for protein-ligand complexes. *J. Med. Chem.* **2006**, *49*, 6177-6196.
(12) Halgren, T. A.; Murphy, R. B.; Friesner, R. A.; Beard, H. S.; Frye, L. L.; Pollard, W. T.; Banks, J. L. Glide: a new approach for rapid, accurate docking and scoring. 2. Enrichment factors in database screening. *J. Med. Chem.* **2004**, *47*, 1750-1759.
(13) Glide, S., LLC, New York, NY. **2021**.
(14) Koeberle, A.; Siemoneit, U.; Buhring, U.; Northoff, H.; Laufer, S.; Albrecht, W.; Werz, O. Licofelone suppresses prostaglandin E2 formation by interference with the inducible microsomal prostaglandin E2 synthase-1. *J. Pharmacol. Exp. Ther.* **2008**, *326*, 975-982.
(15) Fischer, L.; Szellas, D.; Rådmark, O.; Steinhilber, D.; Werz, O. Phosphorylation- and stimulus-dependent inhibition of cellular 5-lipoxygenase activity by nonredox-type inhibitors. *FASEB J.* **2003**, *17*, 1-24.
(16) Steinhilber, D.; Herrmann, T.; Roth, H. J. Separation of lipoxins and leukotrienes from human granulocytes by high-performance liquid chromatography with a Radial-Pak cartridge after extraction with an octadecyl reversed-phase column. *J. Chromatogr.* **1989**, *493*, 361-366.
(17) Albert, D.; Zundorf, I.; Dingermann, T.; Muller, W. E.; Steinhilber, D.; Werz, O. Hyperforin is a dual inhibitor of cyclooxygenase-1 and 5-lipoxygenase. *Biochem. Pharmacol.* **2002**, *64*, 1767-1775.
(18) Garscha, U.; Romp, E.; Pace, S.; Rossi, A.; Temml, V.; Schuster, D.; König, S.; Gerstmeier, J.; Liening, S.; Werner, M.; Atze, H.; Wittmann, S.; Weinigel, C.; Rummler, S.; Scriba, G. K.; Sautebin, L.; Werz, O. Pharmacological profile and efficiency in vivo of diflapolin, the first dual inhibitor of 5-lipoxygenase-activating protein and soluble epoxide hydrolase. *Sci. Rep.* **2017**, *7*, 9398.
(19) Waltenberger, B.; Garscha, U.; Temml, V.; Liers, J.; Werz, O.; Schuster, D.; Stuppner, H. Discovery of Potent Soluble Epoxide Hydrolase (sEH) Inhibitors by Pharmacophore-Based Virtual Screening. *J. Chem. Inf. Model.* **2016**, *56*, 747-762.

(20) Wixtrom, R. N.; Silva, M. H.; Hammock, B. D. Affinity purification of cytosolic epoxide hydrolase using derivatized epoxy-activated Sepharose gels. *Anal. Biochem.* **1988**, *169*, 71-80.
(21) Boyum, A. Isolation of mononuclear cells and granulocytes from human blood. Isolation of monuclear cells by one centrifugation, and of granulocytes by combining centrifugation and sedimentation at 1 g. *Scand. J. Clin. Lab. Invest. Suppl.* **1968**, *97*, 77-89.
(22) Werner, M.; Jordan, P. M.; Romp, E.; Czapka, A.; Rao, Z.; Kretzer, C.; Koeberle, A.; Garscha, U.; Pace, S.; Claesson, H. E.; Serhan, C. N.; Werz, O.; Gerstmeier, J. Targeting biosynthetic networks of the proinflammatory and proresolving lipid metabolome. *FASEB J.* **2019**, *33*, 6140-6153.
(23) Raucci, F.; Saviano, A.; Casillo, G. M.; Guerra-Rodriguez, M.; Mansour, A. A.; Piccolo, M.; Ferraro, M. G.; Panza, E.; Vellecco, V.; Irace, C.; Caso, F.; Scarpa, R.; Mascolo, N.; Alfaifi, M.; Iqbal, A. J.; Maione, F. IL-17-induced inflammation modulates the mPGES-1/PPAR-gamma pathway in monocytes/macrophages. *Br. J. Pharmacol.* **2021**.
(24) Kilkenny, C.; Browne, W.; Cuthill, I. C.; Emerson, M.; Altman, D. G.; Group, N. C. R. R. G. W. Animal research: reporting in vivo experiments: the ARRIVE guidelines. *Br. J. Pharmacol.* **2010**, *160*, 1577-1579.
(25) McGrath, J. C.; Lilley, E. Implementing guidelines on reporting research using animals (ARRIVE etc.): new requirements for publication in BJP. *Br. J. Pharmacol.* **2015**, *172*, 3189-3193.
(26) Bellavita, R.; Raucci, F.; Merlino, F.; Piccolo, M.; Ferraro, M. G.; Irace, C.; Santamaria, R.; Iqbal, A. J.; Novellino, E.; Grieco, P.; Mascolo, N.; Maione, F. Temporin L-derived peptide as a regulator of the acute inflammatory response in zymosan-induced peritonitis. *Biomed. Pharmacother.* **2020**, *123*, 109788.
(27) Chatterjee, B. E.; Yona, S.; Rosignoli, G.; Young, R. E.; Nourshargh, S.; Flower, R. J.; Perretti, M. Annexin 1-deficient neutrophils exhibit enhanced transmigration in vivo and increased responsiveness in vitro. *J. Leukoc. Biol.* **2005**, *78*, 639-646.
(28) Maione, F.; Iqbal, A. J.; Raucci, F.; Letek, M.; Bauer, M.; D'Acquisto, F. Repetitive Exposure of IL-17 Into the Murine Air Pouch Favors the Recruitment of Inflammatory Monocytes and the Release of IL-16 and TREM-1 in the Inflammatory Fluids. *Front. Immunol.* **2018**, *9*, 2752.
(29) Baradaran Rahimi, V.; Rakhshandeh, H.; Raucci, F.; Buono, B.; Shirazinia, R.; Samzadeh Kermani, A.; Maione, F.; Mascolo, N.; Askari, V. R. Anti-Inflammatory and Anti-Oxidant Activity of Portulaca oleracea Extract on LPS-Induced Rat Lung Injury. *Molecules* **2019**, *24*.
(30) Saviano, A.; Casillo, G. M.; Raucci, F.; Pernice, A.; Santarcangelo, C.; Piccolo, M.; Ferraro, M. G.; Ciccone, M.; Sgherbini, A.; Pedretti, N.; Bonvicini, D.; Irace, C.; Daglia, M.; Mascolo, N.; Maione, F. Supplementation with ribonucleotide-based ingredient (Ribodiet(R)) lessens oxidative stress, brain inflammation, and amyloid pathology in a murine model of Alzheimer. *Biomed. Pharmacother.* **2021**, *139*, 111579.
(31) Curtis, M. J.; Bond, R. A.; Spina, D.; Ahluwalia, A.; Alexander, S. P.; Giembycz, M. A.; Gilchrist, A.; Hoyer, D.; Insel, P. A.; Izzo, A. A.; Lawrence, A. J.; MacEwan, D. J.; Moon, L. D.; Wonnacott, S.; Weston, A. H.; McGrath, J. C. Experimental design and analysis and their reporting: new guidance for publication in BJP. *Br. J. Pharmacol.* **2015**, *172*, 3461-3471.
(32) Alexander, S. P. H.; Roberts, R. E.; Broughton, B. R. S.; Sobey, C. G.; George, C. H.; Stanford, S. C.; Cirino, G.; Docherty, J. R.; Giembycz, M. A.; Hoyer, D.; Insel, P. A.; Izzo, A. A.; Ji, Y.; MacEwan, D. J.; Mangum, J.; Wonnacott, S.; Ahluwalia, A. Goals and practicalities of immunoblotting and immunohistochemistry: A guide for submission to the British Journal of Pharmacology. *Br. J. Pharmacol.* **2018**, *175*, 407-411.
(33) George, C. H.; Stanford, S. C.; Alexander, S.; Cirino, G.; Docherty, J. R.; Giembycz, M. A.; Hoyer, D.; Insel, P. A.; Izzo, A. A.; Ji, Y.; MacEwan, D. J.; Sobey, C. G.; Wonnacott, S.; Ahluwalia, A. Updating the guidelines for data transparency in the British Journal of

Pharmacology - data sharing and the use of scatter plots instead of bar charts. *Br. J. Pharmacol.* **2017**, *174*, 2801-2804.
